# Differential Sensitivity of Fruit Pigmentation to Ultraviolet Light between Two Peach Cultivars

**DOI:** 10.3389/fpls.2017.01552

**Published:** 2017-09-08

**Authors:** Yun Zhao, Weiqi Dong, Ke Wang, Bo Zhang, Andrew C. Allan, Kui Lin-Wang, Kunsong Chen, Changjie Xu

**Affiliations:** ^1^Zhejiang Provincial Key Laboratory of Horticultural Plant Integrative Biology, Zhejiang University Hangzhou, China; ^2^Plant and Food Research Auckland, New Zealand; ^3^School of Biological Sciences, University of Auckland Auckland, New Zealand

**Keywords:** peach, anthocyanins, light, glutathione S-transferase, transcription factors

## Abstract

Anthocyanins provide nutritional benefits and are responsible for red coloration in many fruits. Light affects anthocyanin biosynthesis in peach (*Prunus persica*). However, some cultivars show differential sensitivity to light. In the present study, ‘Hujingmilu (HJ),’ a naturally deeply colored cultivar, and ‘Yulu (YL),’ showing low pigmentation, were used to study the mechanism underlying UV-light-induced anthocyanin biosynthesis. Both UVA and UVB induced fruit pigmentation of ‘HJ,’ but ‘YL’ was only sensitive to UVB. Transcriptomic analyses showed over 5000 genes were differentially expressed by pairwise comparisons of RNA libraries isolated from tissue of each cultivar treated with darkness, UVA and UVB. Twenty-three genes related to anthocyanin biosynthesis were identified from the transcriptome data, which were coordinately up-regulated during accumulation of anthocyanins, and down-regulated in the dark. Altered expression of several light receptors, as well as *CONSTITUTIVE PHOTOMORPHOGENIC10* (*COP10*) and *ELONGATED HYPOCOTYL 5 homolog* (*HYH*), and a specific anthocyanin transporter *glutathione S-transferase* (*GST*), in ‘YL’ fruit appears to be responsible for the insensitivity to UVA of this cultivar. Expression profiles of several transcription factors of the families MYB, bHLH, bZIP and NAC were highly correlated with those of the anthocyanin biosynthesis genes. The study provides a valuable overview of the underlying molecular mechanisms of UV-light induced anthocyanin response using peach cultivars with differing light sensitivities.

## Introduction

Pigmentation is important for the ornamental value of plants, and is determined by four categories of compounds: anthocyanins, chlorophylls, carotenoids and betalains (Macheix et al., 1990). Anthocyanins are a group of water-soluble pigments in a wide range of plant tissues, generating reddish, bluish, and purple hues, and as visual signals, play roles in attracting pollinators and seed dispersers (Hichri et al., 2011). Anthocyanins also have crucial biological functions as protective compounds in response to abiotic stresses, such as UV, cold, and drought, and plant defense against herbivores and pathogens (Tsuda, 2012). Furthermore, anthocyanins are reported to have multiple potential health-benefits due to various biological activities like immunomodulatory, antioxidant, cardio-protective, antithrombotic and anti-cancer activities (Clifford et al., 2015; Nemie-Feyissa et al., 2015). In horticulture, fruit color is an important exterior quality for fruit, determining consumers’ acceptance.

Anthocyanins are a subgroup of flavonoids and are stored in the vacuole. The molecular mechanisms underlying anthocyanin accumulation are well established, and the related genes have been characterized in model plants like *Arabidopsis*, snapdragon (*Antirrhinum majus*) and petunia (*Petunia hybrida*) (Winkel-Shirley, 2001). Anthocyanins are produced through phenylpropanoid pathway and genetically determined by both structural and regulatory genes. The structural genes encode enzymes chalcone synthase (CHS), chalcone isomerase (CHI), flavanone 3-hydroxylase (F3H), flavanone 3′-hydroxylase (F3′H), dihydroflavonol 4-reductase (DFR), anthocyanidin synthase (ANS), and UDP glucose-flavonoid 3-*O*-glucosyltransferase (UFGT) (Tanaka et al., 2008). Subsequently, anthocyanin is transported from cytosol into vacuole by proteins such as ATP-binding cassette (ABC), multidrug and toxic compound extrusion (MATE), and glutathione S-transferase (GST) transporters (Koes et al., 2005; Hu et al., 2016). The regulatory genes include MYB, MYC-like basic helix–loop–helix (bHLH) and WD40-repeat proteins (Allan et al., 2008). R2R3 MYBs promote anthocyanin biosynthesis by activating transcription of structural genes, and have been identified in many plants (Lin-Wang et al., 2010; Jaakola, 2013). The bHLH is also an essential component in forming the MYB-bHLH-WD40 (MBW) complex and promoting anthocyanin biosynthesis (Lin-Wang et al., 2010). For example, the interactions between bHLH members TT8, GL3 as well as EGL3 and MYB members PAP1, PAP2, MYB113 as well as MYB114 have been reported in *Arabidopsis* (Gonzalez et al., 2008). The bHLH members participating in regulation of anthocyanin biosynthesis have been identified in some fruit crops like apple (*MdbHLH3/MdbHLH33*, Xie et al., 2012), grape (*VvMYC1*, Hichri et al., 2010), and peach (*PpbHLH3/PpbHLH33*, Ravaglia et al., 2013). The WD40 protein is an intermediate for connecting MYB and bHLH to form MBW complex, which then binds to the promoters of the genes encoding enzymes of the anthocyanin biosynthetic pathway.

Anthocyanin biosynthesis is a complex pathway regulated by plant development and modulated by a suite of environmental factors (Guo et al., 2008). Studies of the molecular mechanisms underlying environmental factors affecting anthocyanin biosynthesis have been made (Lin-Wang et al., 2011; Azuma et al., 2012; Butelli et al., 2012). Light is one of the main environmental factors affecting anthocyanin biosynthesis (Albert et al., 2009). The positive effect of light on elevating fruit anthocyanin content has been reported in many fruit species such as bilberry (Uleberg et al., 2012), Chinese bayberry (*Myrica rubra*, Niu et al., 2010), raspberry (*Rubus idaeus*, Wang et al., 2009) and cranberry (*Vaccinium macrocarpon*, Zhou and Singh, 2004). In addition to light intensity and photoperiod, specific light quality also affects biosynthesis, especially blue and UV light (Li et al., 2013; Ubi et al., 2006). In plants, stress caused by UV light mediates numerous regulatory responses like the enhancement of reactive oxygen species (ROS) and enzymes that repair DNA damage, with effects being dose and genotype dependent. Anthocyanins, as highly effective scavengers of ROS, are reported to be produced by fruits in response to excess UV-light (Zoratti et al., 2014). Postharvest treatments with supplemental UV-light have been performed to increase the contents of beneficial secondary metabolites for improving fruit quality. Blue light and UVC light irradiation treatments on strawberry, at the large green maturity stage, have been reported to increase the antioxidant activity and anthocyanin content (Li et al., 2014; Xu et al., 2014). Anthocyanin enhancement and high expression level of *MdMYBA* in apple skin was found after UVB irradiation (Ban et al., 2007; Peng et al., 2013).

Recently, progress has been made on light perception and signal transduction (Casal, 2013). Specific classes of plant photoreceptors receive light signals to enable plants to sense and respond to light for regulating photomorphogenesis, circadian rhythms and biosynthesis of secondary metabolites, which include phytochromes (PHYs) (red/far-red light receptors), cryptochromes (CRYs), phototropins (PHOTs) (UVA/blue light receptors) and UVR8 (UVB photoreceptor) (Rizzini et al., 2011). Light-induced activation of photoreceptors initiates downstream signal elements like CONSTITUTIVE PHOTOMORPHOGENIC1 (COP1), SUPPRESSOR OF PHYA (SPA), ELONGATED HYPOCOTYL5 (HY5) resulting in light-induced physiological responses (Zhu et al., 2008; Stracke et al., 2010; Lau and Deng, 2012).

In peach, the red pigmentation is due to the presence of anthocyanins, with the predominant component being cyanidin-3-glucoside (Cy-3-glu) (Cheng et al., 2014). *Peace* (MYB-like, Uematsu et al., 2014), *MYB10* (Ravaglia et al., 2013), *Riant* (GST, Cheng et al., 2015), *PpMYB10.4* (Zhou et al., 2014), *BLOOD* (*BL*, a *NAC*) and *PpNAC1* (Zhou et al., 2015) are genes related to anthocyanin accumulation in different organs of peach. Our previous research on peach fruit bagging has shown that a yellow paper bag prevents penetration of UV and blue light, which causes poor peel coloration (Liu et al., 2015). We also observed that coloration varied between cultivars. For un-bagged fruit, ‘Hujingmilu (HJ)’ is naturally deeply colored while ‘Yulu (YL)’ is light colored (Liu et al., 2015). Therefore, the influence of light on anthocyanin accumulation varies in different genetic backgrounds of different cultivars. To further understand the mechanisms of light-induced anthocyanin biosynthesis in peach and the difference in light sensitivity between cultivars, we used RNA-Seq to investigate the transcriptomic differences between two cultivars showing differential light sensitivity under different UV irradiances. Differentially expressed genes (DEGs) and their expression patterns were analyzed, and potential candidate genes responsible for UV-light-mediated anthocyanin biosynthesis were identified. The transcriptome comparisons provide a novel explanation to genotype-related light-dependent anthocyanin accumulation.

## Materials and Methods

### Plant Material and Treatments

‘Hujingmilu (HJ)’ and ‘Yulu (YL)’ peach [*Prunus persica* (L.) Batsch] fruit were collected just before turning stage (100 and 112 days after full bloom respectively for ‘HJ’ and ‘YL’) from an orchard at the Fenghua Peach Research Institute, Zhejiang, China, and were transferred to the laboratory on the day of collection. All peach fruit were bagged pre-harvest at 42 days after full bloom with a commercial yellow paper bag until collection. Fruit of uniform size and maturity were selected as experimental material. The fruit were placed in a chamber at 20°C followed by UVA (315–400 nm, 1000 μw/cm^2^) or UVB (280–315 nm, 58 μw/cm^2^) irradiation for 2 days. The fruit kept in the dark served as the control (CK). For each treatment, 30 fruit were sampled and randomly divided into three biological replicates of 10 fruit each. Peel tissue was sampled, frozen in liquid nitrogen immediately, and then kept at -80°C for further experiments.

### Color Phenotypic Measurement

Fruit surface color was measured by a reflectance spectrophotometer (Hunter Laboratory Mini Scan XE Plus colorimeter). The Commission Internationale de l’Eclairage (CIE) *L^∗^a^∗^b^∗^* color scale was adopted. After recording *L^∗^*, *a^∗^* and *b^∗^*, color index of red grapes (CIRG) was calculated with the formula CIRG = (180-*H*) / (*L^∗^ + C*), while *C* = (*a^∗^*^2^ + *b^∗^*^2^)^0.5^ and *H* = arctan (*b^∗^/a^∗^*) (Carreno et al., 1995; Zhang et al., 2008).

### Extraction and HPLC Analysis of Anthocyanins

Approximately 1 g of peach peel powder was suspended in 5 mL 0.05% HCl in methanol, extracted at 4°C for 12 h, and followed by a centrifugation to collect the supernatant. The residue was extracted for another two times. The supernatants were combined and filtered using a 0.22 μm Millipore membrane, then evaporated in a rotary evaporator at 30°C. The residual was resuspended with 1 ml methanol, filtered with a 0.22 μm Millipore membrane, then analyzed by HPLC using an ZORBAX SB-C18 analytical column (4.6 × 250 mm, 5 μm, Agilent Technologies, Santa Clara, CA, United States). A quantitative analysis of phenolic compounds were analyzed by an Agilent 1260 Liquid Chromatograph equipped with a diode array detector using solvent A (formic acid : water, 5:95, v/v) and solvent B (methanol) with the following gradient: 0–2 min, 5%; 2–7 min, 5–15%; 7–20 min, 15–20%; 20–25 min, 20–27%; 25–32 min, 27%; 32–41 min, 27–35%; and 41.01–43 min, 5% (Cheng et al., 2014). The post-run-time was 5 min. The flow rate was 0.8 ml min^-1^ at 30°C. The wavelength was set at 520 nm. Cyanidin 3-*O*-glucoside chloride purchased from Sigma (CAS:7084) was used as a standard.

### RNA Extraction and Real-time Quantitative RT-PCR (RT-qPCR) Analysis

Total RNA was extracted according to a previously reported CTAB method (Shan et al., 2008; Liu et al., 2015). The extracted RNA was treated with DNA-free DNA Removal kit (Invitrogen, Life Technologies, Camarillo, CA, United States). For each sample, one microgram of total RNA was used for cDNA synthesis with iScript cDNA synthesis kit (Bio-Rad, Hercules, CA, United States). Real-time PCR was carried out with SsoFast EvaGreen Supermix kit (Bio-Rad, Hercules, CA, United States) using a CFX96 instrument (Bio-Rad, Hercules, CA, United States) under the following parameters: 95°C for 3 min and 45 cycles of 95°C for 10 s, 60°C for 30 s, and then 95°C for 10 s followed by a continuous increase from 65°C to 95°C with a ramp rate of 0.5°C/s for dissociation curve analysis (Yin et al., 2012). Each reaction (final volume, 20 μL) contained 2× real-time PCR mix (10 μL), primer (1 μL, 10 μM, Supplementary Table S1), cDNA template (2 μL), and RNase free water (6 μL). Peach gene *PpTEF2* (Translation Elongation Factor 2, JQ732180) was used as the reference gene for normalization (Tong et al., 2009).

### Library Construction, Transcriptome Sequencing and Data Analysis

Total RNA was extracted from peach fruit peel as mentioned above. The purity and integrity analysis were measured with RNA Nano 6000 Assay Kit of the Bioanalyzer 2100 system (Agilent Technologies, Santa Clara, CA, United States). mRNA enrichment and fragmentation, second-strand cDNA synthesis, size selection, PCR amplification and subsequent sequencing was performed by staff at Novogene Bioinformatics Technology Co. Ltd. (Beijing, China) with an Illumina HiSeq 2500 platform.

RNA-Seq was performed and 50 bp single-end reads were generated. Raw data (raw reads) were then filtered via removing the adapter reads and low quality reads (the reads with N percentage over 5% or those containing over 20% nucleotides in read with *Q*-value below 10). Q20, Q30 and GC content were estimated to ensure clean reads with high quality for further analysis. Index of the reference genome obtained from the genome database (GDR^1^) was built with Bowtie v2.0.6. TopHat v2.0.12 was used for mapping single-end clean reads to the reference genome. The read number mapped to each gene was counted by HTSeq v0.6.1.

Gene expression level was normalized by calculating FPKM (expected number of Fragments Per Kilobase of transcript sequence per Millions base pairs sequenced). DESeq R package (1.10.1) was used to conduct differential expression analysis of samples. DEGs between treated and CK samples were identified if adjusted *P* ≤ 0.05 and |log_2_FoldChange|≥ 1. Gene Ontology (GO) and KEGG Pathways (Kyoto Encyclopedia of Genes and Genomes^2^) enrichment analysis were further conducted on DEGs respectively by the GOseq R packages and KOBAS 2.0 software.

### Phylogenetic Analysis

Deduced amino acid sequences were aligned with the ClustalX (1.81) and optimized in GeneDoc. The phylogenetic tree was constructed after alignment with MEGA v6.06 using the neighbor-joining method and 1000 bootstrap replicates.

### Correlation Analysis of Structural Genes and Transcription Factors

Differentially expressed transcription factors were selected from transcriptome data (|log_2_FoldChange|≥ 1 and adjusted *P* ≤ 0.05). Based on FPKM value, correlation analysis of differentially expressed transcription factors and anthocyanin structural genes was conducted by MetaboAnalyst^3^. A significant correlation was considered to exist between transcription factors and anthocyanin structural genes with *P* ≤ 0.05 as the thresholds.

## Results

### Coloration and Anthocyanin Levels in the Peel of Peach Fruit after UV-Light Treatment

**FIGURE 1:**
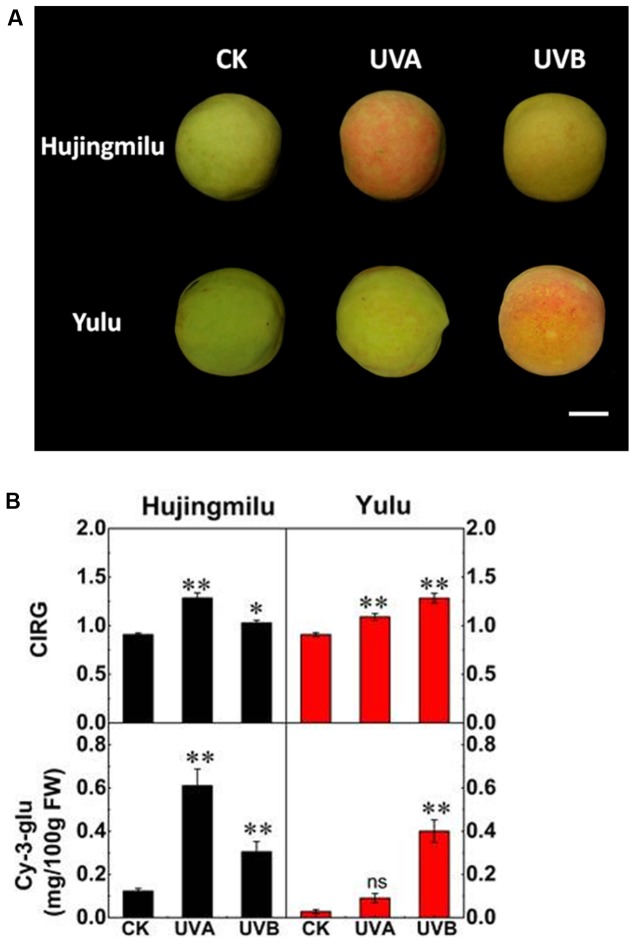
Effects of UV exposure on peel color and anthocyanin content in peach. **(A)** Representative photographs of peach fruit under dark (CK), UVA and UVB conditions for 2 days. **(B)** The color index of red grapes (CIRG) values and cyanidin-3-glucoside (Cy-3-glu) content in peach peel. The vertical bars represent the standard error of three biological replicates. White bar = 23 cm. Data marked with ^∗∗^ and ns indicate *P* < 0.01 and not significant, respectively.

The color of peach peel changed to differing extents after UV-light treatment. Color differences were measured and presented as color index of red grapes (CIRG) values, and Cy-3-glu was measured after 2 days of treatment. The ‘HJ’ peel remained light green in dark conditions but reddened when treated with UV-light for 2 days, with UVA having a stronger effect on coloration than UVB (**Figure 1A**). Cy-3-glu levels reached to 0.61 mg/100 g FW under UVA irradiation and 0.31 mg/100 g FW under UVB irradiation respectively (3–4 fold higher than CK). The ‘YL’ cultivar reddened only under UVB irradiation, with anthocyanin content increasing to 0.4 mg/100 g FW, a 14-fold increase compared to CK (**Figure 1B**). The ‘YL’ fruit subjected to UVA exposure still remained green and did not accumulate significant amounts of anthocyanin. In general, Cy-3-glu contents matched well the visual red appearance of the fruit.

### Transcriptome Sequencing

Six cDNA libraries were constructed from the total RNA of ‘HJ’ and ‘YL’ peach under dark (CK), UVA and UVB condition. Each library generated over 13 million reads (Supplementary Table S1) (data available at the NCBI SRA database with ID SRP116640)^4^. The sequences were trimmed for adapter sequences and low quality reads filtered out. In ‘HJ,’ the consensus assembly from the three treatments generated an average of 0.71 gigabyte clean bases (Q20 > 98.8%; Q30 > 97.6% with sequencing error rates lower than 1%), and 89.1% of total reads were uniquely mapped to the peach genome. For ‘YL,’ an average of 6.71 gigabyte clean reads (Q20 > 97.3%; Q30 > 93.1%) were obtained, and 88.8% of total reads were uniquely mapped to the peach genome. The high reproducibility of the data was indicated by high correlation coefficients (>0.97, Supplementary Figure S1) between biological replicates. The quality of unigene data was sufficient for further analysis.

### Analysis of Differentially Expressed Genes (DEGs) between ‘HJ’ and ‘YL’

**FIGURE 2:**
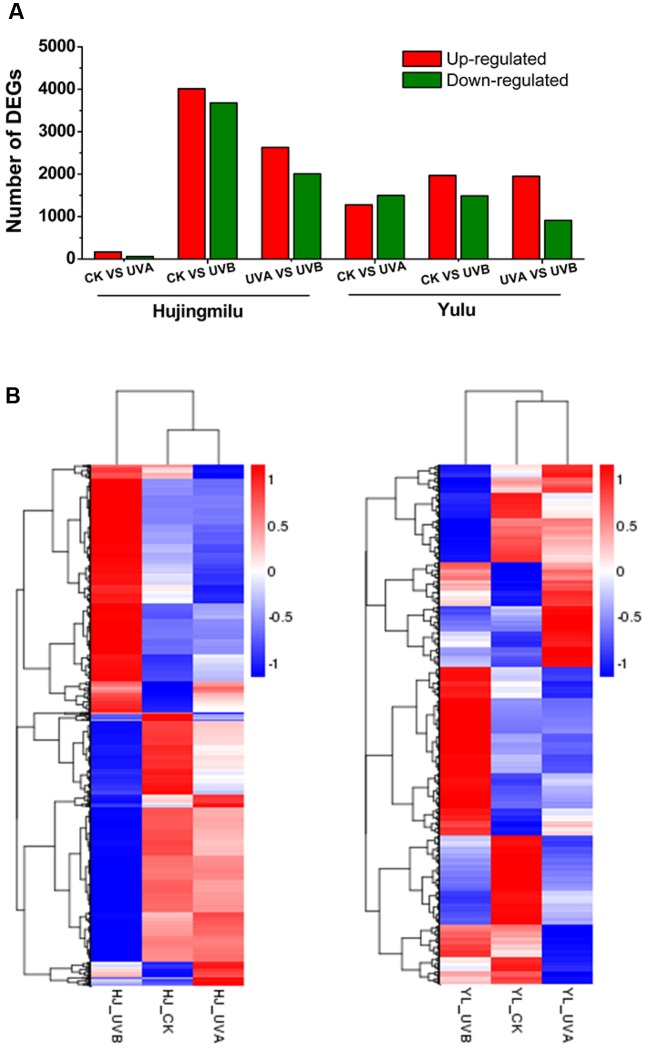
Overview of transcriptome of ‘Hujingmilu’ (‘HJ’) and ‘Yulu’ (‘YL’) peach fruit under different light conditions (Dark (CK), UVA and UVB). **(A)** Number of differentially expressed genes (DEGs) (|log_2_FoldChange|≥ 1 and adjusted *P* < 0.05) identified by pairwise comparison under dark, UVA and UVB conditions of each cultivar for 2 days. **(B)** Hierarchy clustering of DEGs across the different treatments. The rows in the heatmap represent genes, and the columns indicate samples. The colors of heatmap cells indicate scaled expression level of genes (log_2_ FPKM) across different samples. The color gradient, ranging from blue, through white, to red represents low, middle and high values of gene expression.

Differentially expressed genes were analyzed to investigate the underlying mechanisms for differential accumulation of anthocyanins between the two cultivars. Expression levels were measured as FPKM. Differences in gene expression in the peel under three treatments were assessed using pairwise comparisons of the three libraries (UVA-VS-CK, UVB-VS-CK, and UVB-VS-UVA) of each cultivar with the expression fold |log_2_FoldChange|≥ 1 and adjusted *P* ≤ 0.05 as the thresholds. In total 7996 DEGs were identified in cv. ‘HJ’ between treatments; 225 DEGs (166 upregulated and 59 downregulated) were identified between UVA and CK, 7690 DEGs (4012 upregulated and 3678 downregulated) between UVB and CK, and 4635 DEGs (2629 up-regulated and 2006 downregulated) between UVB and UVA (**Figure 2A**). For cv. ‘YL,’ a total of 5647 DEGs were differentially expressed between comparisons, with 2773 (1276 upregulated and 1497 downregulated), 3457 (1969 upregulated and 1488 downregulated), and 2858 (1950 upregulated and 908 downregulated) DEGs between UVA and CK, UVB and CK, UVB and UVA, respectively. The largest number of DEGs occurred with UVB and CK in both cultivars. Hierarchy cluster analysis (HCA) was performed (**Figure 2B**); UV irradiation promoted expression of clusters of genes while inhibiting other clusters. For both cultivars, DEGs of fruit under UVA were more closely clustered with CK rather than DEGs of fruit under UVB (**Figure 2B**), consistent with the data on the number of DEGs (**Figure 2A**).

All DEGs were searched against reference pathways in the KEGG database^5^. An overview of the functional enrichment of pathways between treatments and cultivars provides a global description of the biological pathway enriched in different transcripts (Supplementary Figures S2, S3). The data generated from comparing UVA with CK in ‘HJ’ reveal that flavonoid biosynthesis (10 DEGs/39 background genes) was the most enriched pathway, followed by circadian rhythm (7 DEGs/35 background genes), DNA replication (6 DEGs/47 background genes), and photosynthesis (6 DEGs/68 background genes) using adjusted *P* ≤ 0.05 as thresholds. The DEGs participating in the flavonoid biosynthesis category included *cinnamic acid 4-hydroxylase* (*C4H*), *CHS, F3H, F3′H, DFR*, and *flavonol synthase* (*FLS*). A total of 18 DEGs, including gene family members of *C4H*, *CHS*, *CHI*, *F3H*, *F3’H*, *DFR*, *ANS*, *leucoanthocyanidin reductase* (*LAR*), *anthocyanidin reductase* (*ANR*), were assigned to flavonoid biosynthesis category for UVB-VS-CK and 15 DEGs (including *C4H*, *CHS*, *CHI*, *F3’H*, *FLS*, *LAR*, *ANR)* for UVB-VS-UVA. In cultivar ‘YL’ in flavonoid biosynthesis, 10 DEGs (UVA vs. CK, including *CHS*, *F3H*, *ANS*), 7 DEGs (UVB vs. CK, including *CHS*, *CHI*, *ANS*, *LAR*) and 9 DEGs (UVB vs. UVA, including *CHS*, *CHI*, *F3H*, *F3’H*, *LAR*) were found in pairwise comparisons between the three libraries.

**FIGURE 3:**
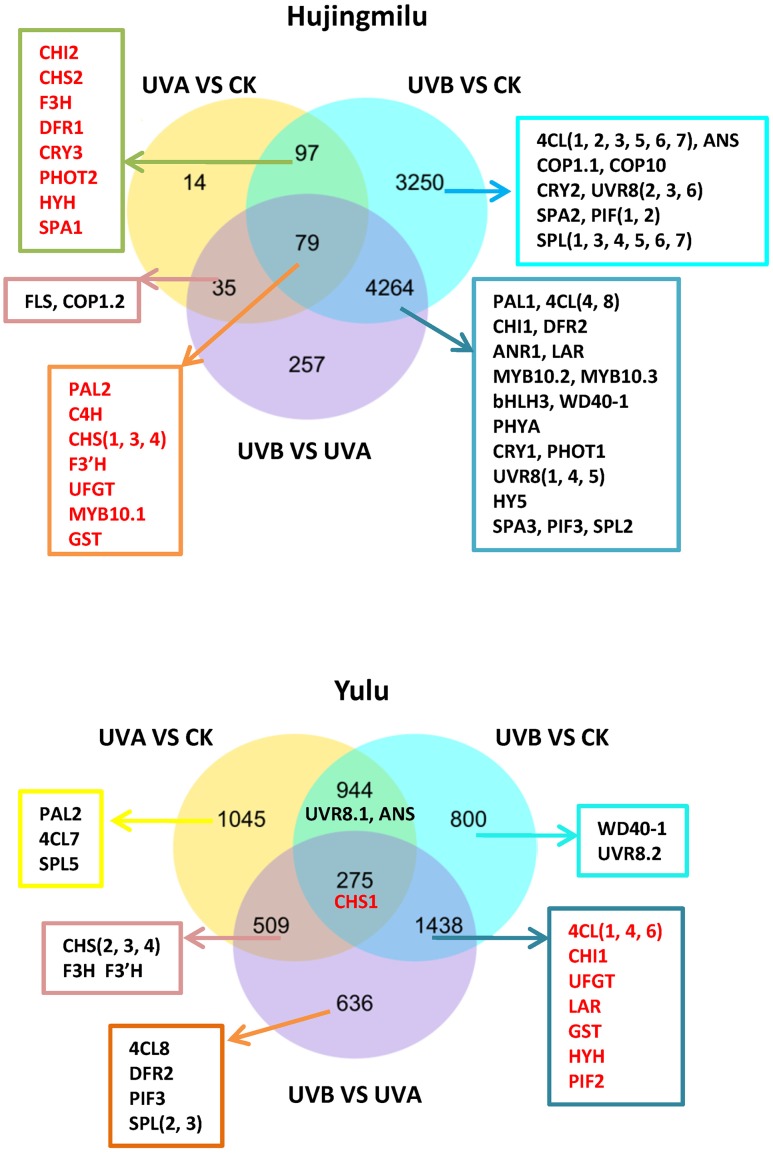
Venn diagrams illustrating the number of differentially expressed genes (DEGs) revealed by paired comparison between dark (CK), UVA and UVB treatments in peach fruit. 4CL, 4-coumarate coenzyme A ligase; ANR, anthocyanidin reductase; ANS, anthocyanidin synthase/leucoanthocyanidin dioxygenase; bHLH, basic helix–loop–helix; C4H, cinnamic acid 4-hydroxylase; CHS, chalcone synthase; CHI, chalcone isomerase; CRY, cryptochrome; COP1, constitutive photomorphogenic 1; COP10, constitutive photomorphogenic 10; DFR, dihydroflavonol-4-reductase; F3H, flavanone 3-hydroxylase; F3′H, flavanone 3′-hydroxylase; FLS, flavonol synthase; GST, glutathione S-transferase; HY5, ELONGATED HYPOCOTYL 5; HYH, HY5 homolog; LAR, leucoanthocyanidin reductase; PAL, phenylalanine ammonia lyase; PHYA, phytochrome A; PHOT, phototropin; PIF, phytochrome-interacting basic helix-loop-helix transcription factors; SPA, suppressor of PHYA; SPL, SQUAMOSA promoter-binding protein-like; UFGT, UDP-glucose: flavonoid-3-*O*-glucosyltransferase; UVR8, UV RESISTANCE LOCUS 8.

The DEGs were visualized in Venn diagrams for an overall observation of expression patterns. Of the 7996 and 5647 DEGs discovered in the ‘HJ’ and ‘YL’ libraries respectively, 79 (0.99%) and 275 (4.87%) DEGs were significantly differentially expressed in all the three pairwise comparisons for both cultivars (**Figure 3**). Over 60 anthocyanin structural genes as well as photoreceptor and light signal transduction genes were found to be differentially expressed in ‘HJ’, but less than 30 genes were seen in ‘YL.’ Both UVA and UVB treatments can induce a DEG set leading to anthocyanin accumulation in ‘HJ’ peel, but only UVB seemed to have this effect on ‘YL.’ The 176 (97 + 79) DEGs (the common difference between CK and UVA/UVB) of ‘HJ’ and 1713 (275+1438) DEGs (the common difference between CK/UVA and UVB) of ‘YL’ were further analyzed. In ‘HJ’ fruit, DEGs included anthocyanin structural genes (*PAL2*, *C4H*, *CHSs*, *CHI2*, *F3H*, *F3′H*, *DFR1*, *UFGT*), the specific anthocyanin transporter gene *GST*, the transcription factor *MYB10.1*, and three light signal transduction genes [*CRY3*, *PHOT2*, *HY5 homolog* (*HYH*), *SPA1*]. Among the 1713 DEGs of ‘YL’ fruit, the anthocyanin structural genes *4CLs*, *CHS1*, *CHI1*, *UFGT*, the specific anthocyanin transporter gene *GST*, and two light signal transduction genes [*HYH*, *phytochrome-interacting basic helix-loop-helix transcription factors 2* (*PIF2*)] were present.

### Expression of Genes Associated with Anthocyanin Metabolic Pathway

**FIGURE 4:**
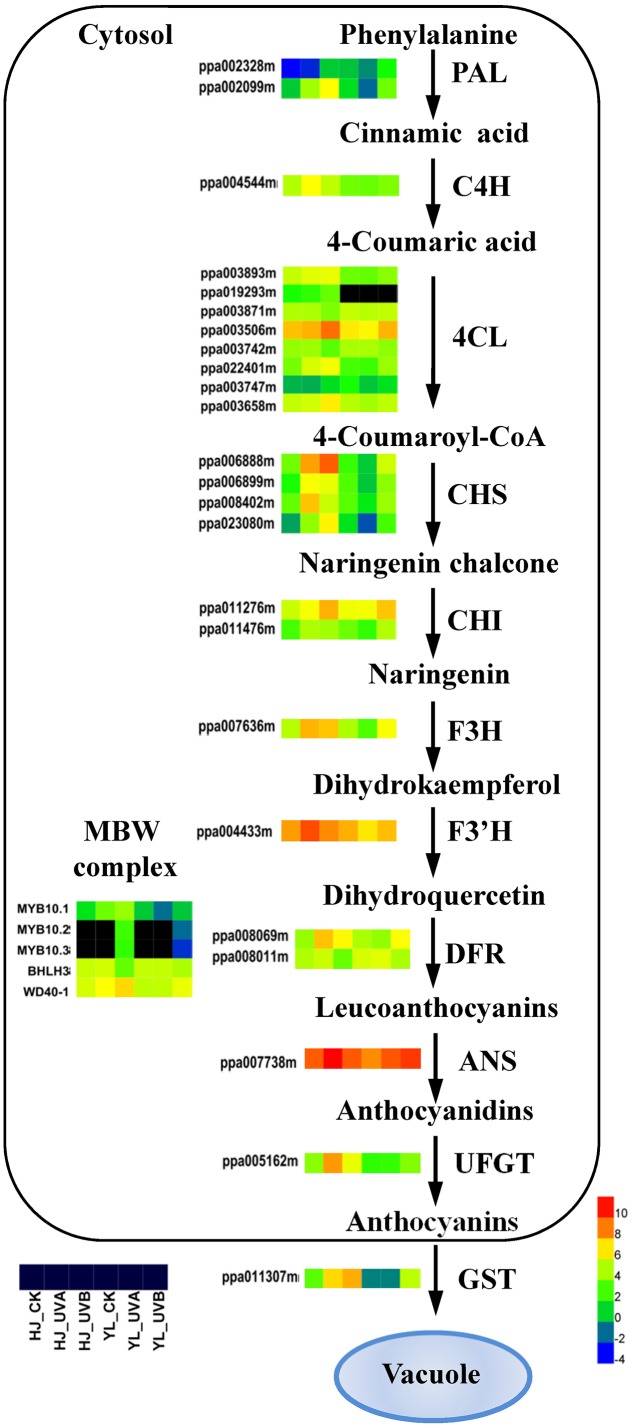
Effects of light treatment on expression of structural genes related to anthocyanin biosynthesis in peach. Enzyme names, gene IDs and expression patterns are indicated on the side of each step for anthocyanin biosynthesis/transport. For each gene, the first three squares represent ‘Hujingmilu’ (‘HJ’) and the last three squares represent ‘Yulu’ (‘YL’). Different colors represent different expression levels. The color gradient with eight different colors from blue (–4) to red (10) corresponds to transcript levels from low to high, with the values representing the log_2_ (FPKM) values and the black color means FPKM = 0. ANS, anthocyanidin synthase/leucoanthocyanidin dioxygenase; bHLH, basic helix–loop–helix; C4H, cinnamic acid 4-hydroxylase; CHS, chalcone synthase; CHI, chalcone isomerase; DFR, dihydroflavonol-4-reductase; F3H, flavanone 3-hydroxylase; F3′H, flavanone 3′-hydroxylase; FPKM, expected number of Fragments Per Kilobase of transcript sequence per Millions base pairs sequenced; GST, glutathione S-transferase; PAL, phenylalanine ammonia lyase; UFGT, UDP-glucose: flavonoid-3-*O*-glucosyltransferase; 4CL, 4-coumarate coenzyme A ligase.

A pathway of anthocyanin biosynthesis describing the expression of all DEGs was showed (**Figure 4**). *PAL* catalyzes the first step of phenylpropanoid pathway. Expression of two *PALs* (ppa002328m, ppa002099m) and one *C4H* (ppa004544m) was low in green peel (HJ_CK, YL_CK, YL_UVA) but was high in the red peel (HJ_UVA, HJ_UVB, YL_UVB). Transcripts corresponding to seven *4CL* genes showed distinct expression patterns. Profiles of four *4CL* gene models were highly expressed (ppa003893m, ppa003506m, ppa022401m, and ppa003658m) and consistent with the accumulation of anthocyanin in the two cultivars. Four *CHS* genes (ppa006888m, ppa006899m, ppa008402m, ppa023080m) showed a similar expression pattern which paralleled accumulation of anthocyanins. Two *CHI* genes (ppa011276m, ppa011476m) and one *F3H* gene (ppa007636m) showed a similar expression trend as *CHS*. The expression patterns of late stage genes, two *DFRs* (ppa008069m and ppa008011m), one *ANS* (ppa007738m) and one *UFGT* (ppa005162m) were also correlated with anthocyanin content. The *ANS* transcript reached highest expression with a FPKM of over 2,000 under UVA in ‘HJ’, almost 15 fold higher than CK (**Figure 4** and Supplementary Table S4).

The expression profile of most structural genes showed distinctive patterns in the two cultivars, especially following UVA treatment. In ‘HJ’, expressions of most structural genes were significantly upregulated, by 2–45-fold, in response to both UVA and UVB. In ‘YL,’ most structural genes showed higher transcript abundance following UVB exposure, e.g., 3–7 fold higher for *CHS*, *F3H*, *DFR*, *ANS* and *UFGT*, but were not affected by UVA (except for ANS and DFR2) (**Figure 4** and Supplementary Table S4). In CK fruit the expression of ANS and UFGT in ‘HJ’ was around two and three times of that in ‘YL’ (**Figure 4** and Supplementary Table S4).

The known MBW complex regulatory genes in peach, related to anthocyanin biosynthetic pathway, includes *PpMYB10.1/2/3* (ppa026640m/ppa016711m/ppa020385m), *PpbHLH3* (ppa002884m) and *PpWD40-1* (ppa008187m). For CK, the expression of *PpMYB10.1* was around one-third lower in ‘YL’ than in ‘HJ’. Following UVA treatment, the expression of *PpMYB10.1* was increased by around 10 times in ‘HJ’ but was reduced to one-third in ‘YL’, while following UVB treatment, the expression of the three *PpMYB10s* and *PpWD40-1* were both induced in ‘HJ’ and ‘YL.’ However, the expression of *PpbHLH3* was reduced in ‘HJ’ and ‘YL following UVB treatment (**Figure 4** and Supplementary Table S4).

To validate the accuracy of gene expression profiles, we selected 27 anthocyanin biosynthesis genes from the two cultivars at different treatments and analyzed the gene expression using RT-qPCR. Linear regression analysis displayed a general correlation coefficient of 0.831^∗∗^ for transcript abundance data from RT-qPCR with transcriptome data (Supplementary Figure S4). This suggested that transcriptome data was reliable and could be used for expression analysis for other genes. The IDs, primers, real-time PCR value, and FPKM value were shown in Supplementary Tables S2, S3.

### *PpGST1* Correlated with Anthocyanin Accumulation

**FIGURE 5:**
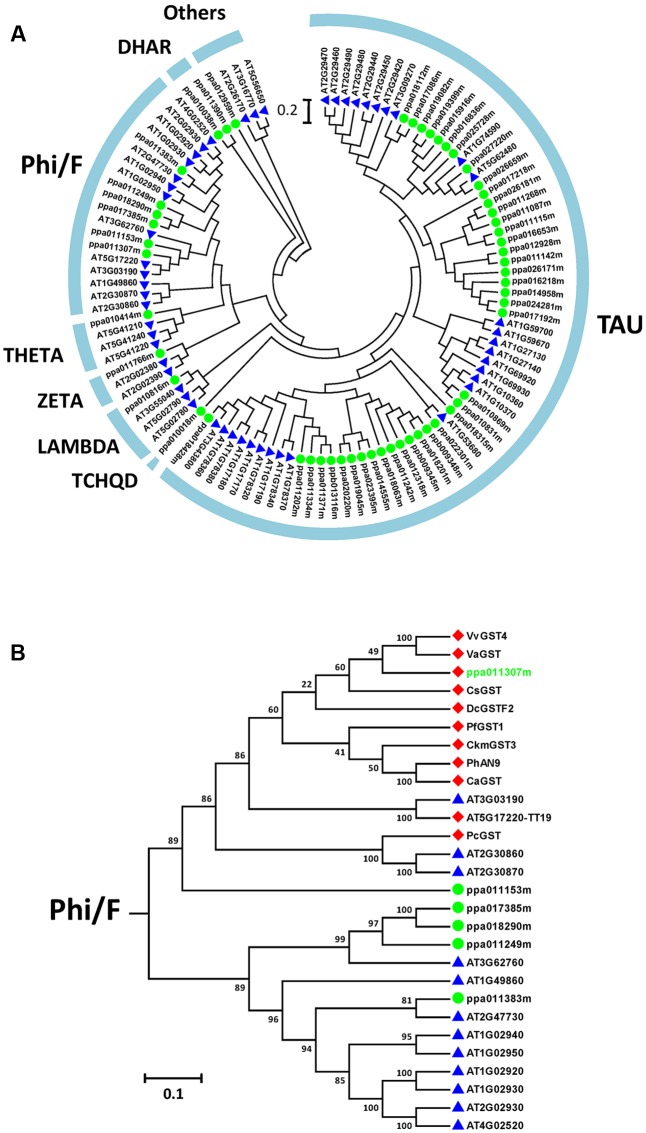
Phylogenetic tree of plant glutathione S-transferases (GSTs). **(A)** Phylogenetic tree of 52 GSTs from *Arabidopsis* (blue triangles) and 54 GSTs from peach (green dots). **(B)** The Phi/F group GSTs from *Arabidopsis*, peach, and those related to anthocyanin biosynthesis in other plants. Red diamonds, published anthocyanin-related GSTs as well as a peach candidate; green dots, other phi-type GSTs of peach; blue triangles, other Phi-type GSTs of *Arabidopsis*. Anthocyanin-related GSTs: AtTT19 (*Arabidopsis thaliana*, OAO91277), CaGST (*Capsicum annuum*, XP_016562106), CkmGST3 (*Cyclamen*, BAM14584), CsGST (*Citrus sinensis*, NP_001275781), DcGSTF2 (*Dianthus caryophyllus*, BAM21533), PcGST (*Pyrus communis*, ABI79308), PfGST1 (*Perilla frutescens*, BAG14300), PhAN9 (*Petunia hybrida*, CAA68993), VaGST (*Vitis amurensis*, ACN38271), VvGST4 (*Vitis vinifera*, AAX81329), ZmBZ2 (*Zea mays*, CAA57496).

Among the DEGs identified from responses in both cultivars, *PpGST1* (ppa011307m) was observed (**Figure 3**). GST is a large gene family which catalyzes the conjugation of the reduced form of glutathione (Wu et al., 2014). Some GST members are indispensable for anthocyanin transport such as grapevine *VvGST4*, citrus *CsGST* (Licciardello et al., 2008, 2014), carnation *DcGSTF2*, perilla *PfGST*, and petunia *PhAN9*. A phylogenetic tree was constructed between *Arabidopsis GST* family members and peach candidate *GST* family members (**Figure 5A**). It was shown that 54 candidate GST family members from peach transcriptome dataset were clustered into seven groups (**Figure 5A**).

**FIGURE 6:**
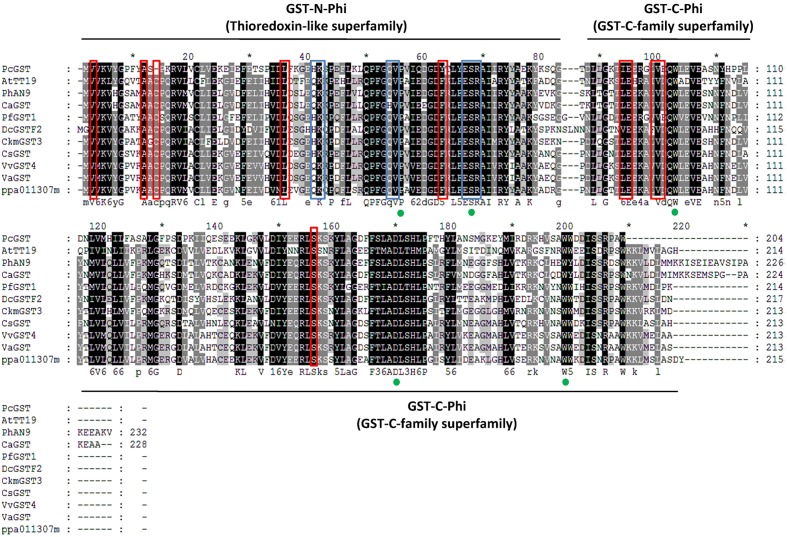
Protein sequence alignment of PpGST (ppa011307m) and other anthocyanin-related GSTs. Numbers in the alignments show amino acid positions. Green dots indicate amino acid residues that were suggested previously to be high-homology sites in the GST family (Alfenito et al., 1998). Blue boxes indicate the regions that are involved in glutathione binding in AtGSTF2 (Reinemer et al., 1996). Red boxes indicate anthocyanin-related GST-specific amino acid residues in the alignment (Kitamura et al., 2012).

When all *Arabidopsis* and peach *GST* family members were clustered with another ten published anthocyanin-related *GSTs* from other dicotyledonous plants, it was found that *PpGST1* (ppa011307m) clustered in the same group (the Phi/F group). The Phi/F group, including *PpGST1* (ppa011307m), are the most likely GSTs involved in vacuolar accumulation of anthocyanin (**Figure 5B**). An alignment of predicted PpGST protein and previously known anthocyanin-related GSTs revealed that all these GSTs contained a GST-N-Phi (Thioredoxin-like superfamily) domain and a GST-C-Phi (GST-C-family superfamily) domain as other Phi-type GSTs of *Arabidopsis* (**Figure 6**). Several conserved amino acid residues were observed in a multiple alignment including high-homology sites of GST family, glutathione binding sites and anthocyanin-related GST-specific sites.

The transcript level of *PpGST1* was differentially expressed and correlated with the accumulation of anthocyanin in two cultivars following UV irradiation. It was up-regulated 12-fold (after UVA treatment) and 22-fold (after UVB treatment) in UV-light-exposed ‘HJ’ compared with CK; while being up-regulated 50-fold in UVB-exposed ‘YL’ compared with both CK and UVA. Strikingly, expression of *PpGST1* was not affected by UVA treatment in ‘YL.’ The expression was also higher in CK fruit of ‘HJ’ than ‘YL’ (**Figure 4** and Supplementary Table S4).

### Expression of Photoreceptor and Light Signal Transduction Related Genes

**FIGURE 7:**
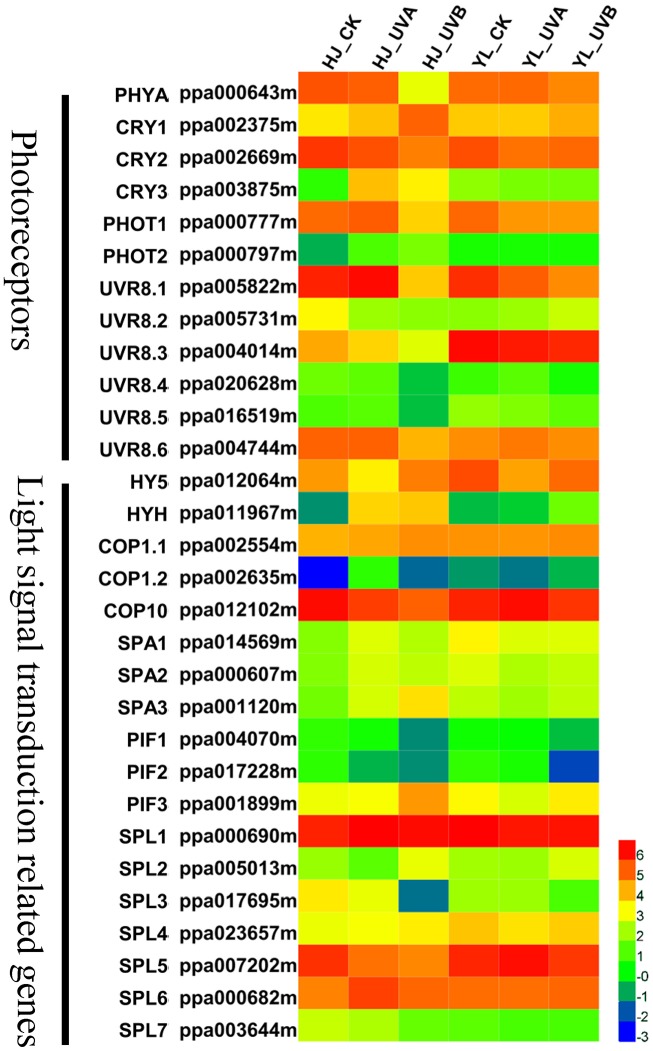
Heatmap representation of the expression patterns of photoreceptors and light signal transduction related genes. Rows and columns in the heat map represent genes and samples collected. The color scale at the right represents the log-transformed FPKM values. CRY1, cryptochrome 1; CRY2, cryptochrome 2; CRY3, cryptochrome 3; COP1, constitutive photomorphogenic 1; COP10, constitutive photomorphogenic 10; FPKM, expected number of Fragments Per Kilobase of transcript sequence per Millions base pairs sequenced; HY5, ELONGATED HYPOCOTYL 5; HYH, HY5 homolog; PHYA, phytochrome A; PHOT1, phototropin 1; PHOT2, phototropin 2; PIFs, phytochrome-interacting basic helix-loop-helix transcription factors; SPA, suppressor of PHYA; SPL, SQUAMOSA promoter-binding protein-like; UVR8, UV RESISTANCE LOCUS 8.

Light photoreceptors include four groups: PHYs, CRY, PHOTs, and UVR8 (Rizzini et al., 2011; Casal, 2013). CRY, PHOTs, and UVR8 are photoreceptors mediating photomorphogenic responses to UVA and UVB respectively. After UV-light irradiation, the expression pattern of *PpPHYA* (ppa000643m) declined in both cultivars, especially following UVB treatment. *PpCRY1* (ppa002375m), *PpCRY2* (ppa002669m), *PpCRY3* (ppa003875m) and two *PpPHOTs* (ppa000777m, ppa000797m) were observed in transcriptome data. The expression patterns of *PpCRY1* and *PpCRY3* as well as *PpPHOT1* and *PpPHOT2* increased to varying degrees when the ‘HJ’ fruit were exposed to UVA, especially *PpCRY3*. In the ‘YL’ cultivar, the expression levels of all *PpCRYs* and *PpPHOTs* were lower or not significantly changed compared to CK, suggesting one possible mechanism why ‘YL’ is insensitive to UVA. Six genes putatively encoding PpUVR8 (ppa005822m, ppa005731m, ppa004014m, ppa020628m, ppa016519m, ppa004744m) were identified, and generally, the expression of these *PpUVR8s* was inhibited following UVB treatment in both cultivars (**Figure 7**).

Located downstream of the photoreceptors, HY5 is a positive central modulator for light signaling coordination and anthocyanin biosynthesis regulation, and is a target of COP1 for degradation under dark condition (Osterlund et al., 2000). Here we identified *PpHY5* (ppa012064m) and *PpHYH* (ppa011967m) genes through a phylogenetic tree of *Arabidopsis* and peach bZIP family members (Supplementary Figure S5). After UV light exposure, the transcript level of *PpHY5* decreased under UVA treatment for ‘HJ’ and UVB treatment for ‘YL,’ which was poorly correlated with anthocyanin levels in both cultivars (**Figure 7**). However, the transcript abundance of *PpHYH* was consistent with anthocyanin content. UVA and UVB increased *PpHYH* expression by over 35 fold in ‘HJ,’ and UVB induced expression of *PpHYH* by 4 fold in ‘YL’ compared to CK and UVA.

The Ubiquitin E3 ligase COP1 is a master repressor in light-induced plant development processes such as photomorphogenesis, DNA repair and anthocyanin biosynthesis via directing protein degradation. In our study, two genes putatively encoding COP1 in peach were identified, and these two COP1 proteins both contain a conserved RING domain and WD-40 repeat motifs (Supplementary Figure S6). For both cultivars, as compared with CK fruit, the transcription levels of *PpCOP1.1* (ppa002554m, the gene family member with the highest expression) were not differentially expressed in the UV-light treated fruit (**Figure 7** and Supplementary Table S5). COP10, a negative regulator of photomorphogenesis, is indispensable in COP1-mediated HY5 degradation process. Different from ubiquitin E3 ligase COP1 members, it contains an ubiquitin-conjugating (UBC) motif rather than a WD-40 repeat motif, belonging to ubiquitin E2 variant (UEV) protein family (Sanch et al., 1998). *PpCOP10* (ppa012102m) was identified to be a candidate member participating in regulation of photomorphogenesis (Supplementary Figure S6). The transcriptional levels of *PpCOP10* were generally high and showed an opposite trend with anthocyanin content. The transcription level of *PpCOP10* decreased after exposure to UVA and UVB in ‘HJ.’ In ‘YL’ peach, its expression was higher in uncolored fruit of CK and UVA treatment compared with red-colored fruit of UVB treatment (**Figure 7**).

In addition, SPA, PIF and SQUAMOSA promoter-binding protein-like (SPL) are also important negative elements in the light signaling pathway synergistically repressing photomorphogenesis with COP1. The expression profiles of two *PpPIFs* (ppa004070m, ppa017228m) and three *PpSPLs* (ppa017695m, ppa007202m, ppa003644m) were similar to the transcription level of *PpCOP10* (**Figure 7**). Three putative *PpSPAs* (ppa014569m, ppa000607m, ppa001120m) were not correlated with the anthocyanin content in fruit samples.

### Prediction of Candidate Transcription Factors Related to Anthocyanin Biosynthesis

Transcription factors (TFs) play key roles in regulating anthocyanin biosynthesis. Among all DEGs of ‘HJ’ (Supplementary Figure S7), 426 differentially expressed TFs belonging to 48 families were found, mainly including MYBs, MYB-related, bHLH, bZIP, C2H2, NAM/ATAF/CUC (NAC), WRKY and ethylene response factor (ERF). In ‘YL’, 425 TFs, from 61 gene families, were identified. The expression of genes in major TF families is presented in Supplementary Figure S7.

A transcript abundance correlation analysis was used to identify candidate differentially expressed TFs co-expressed with anthocyanin biosynthesis structural genes. The expression levels of 21 transcription factors were highly correlated with those of structural genes in ‘HJ’ and ‘YL’ based on correlation and clustering analysis (Supplementary Figure S7 and **Table 1**). A total of eight differentially expressed transcription factor genes annotated as *MYB* (ppa026640m, ppa016711m, ppa020385m, ppa004560m, ppa008450m, ppa015973m, ppa023812m, ppa017136m) showed a significant correlation with anthocyanin biosynthetic genes for ‘HJ’ and ‘YL.’ Among these, ppa026640m (*PpMYB10.1*), ppa016711m (*PpMYB10.2*), ppa020385m (*PpMYB10.3*), homologous to *AtMYB113* (Gonzalez et al., 2008), were differentially expressed only in ‘HJ.’ *PpMYB10.2* and *PpMYB10.3* only responded to UVB, while the ppa004560m (homologous to *AtMYB12*) only responded to UVA. Homologs of *ZmMYBC1* (ppa023812m and ppa017136m) had a high correlation to anthocyanin accumulation in ‘YL’ cultivar. Five *PpbHLHs* (ppa002884m, ppa004070m, ppa017228m, ppa010134m, ppa006295m) were correlated with the anthocyanin biosynthetic pathway. Among these, ppa002884m (PpbHLH3), homologous to AtTT8, was reported as a positive regulator for anthocyanin biosynthesis in peach fruit, and two PpPIFs (ppa004070m, ppa017228m) may participate in light signal transduction as described above (Rahim et al., 2014). In our study, three PpbZIP TFs (ppa009024m, ppa011967m, ppa005585m) were identified. These genes were all upregulated after exposure of fruit to UV-light. The expression of bZIP gene *PpHYH* (ppa011967m, *homologous to HY5*) was consistent with the anthocyanin accumulation. A positive correlation between expression of five *PpNACs* (ppa009438m, ppa009380m, ppa026582m, ppa007883m, ppa025263m) and that of structural genes was observed. With the exception of ppa007883m (homologous to *AtNAC100*), the remaining four *NACs* all had a strong response to UVB irradiation (Supplementary Figure S7).

**Table 1:** List of differentially expressed transcription factors highly correlated with structural genes involved in anthocyanin metabolism.

Peach ID	TF family	Cultivar	Peach/model plant	Description in model plant
ppa026640m	MYB	HJ	PpMYB10.1/AtMYB113	Involved in regulation of anthocyanin biosynthesis. Affects the expression of enzymes involved in later steps of anthocyanin biosynthesis.
ppa016711m	MYB	HJ	PpMYB10.2/AtMYB113	
ppa020385m	MYB	HJ	PpMYB10.3/AtMYB113	
ppa004560m	MYB	HJ	PpMYB12/AtMYB12	Regulates flavonoid accumulation and abiotic stress tolerance.
ppa008450m	MYB	HJ	PpMYB44/AtMYBr1	Involved in mediating plant responses to a variety of abiotic stimuli.
ppa015973m	MYB	YL	PpMYB108/AtMYB2	Regulates the expression of salt- and dehydration-responsive genes.
ppa023812m	MYB	YL	PpTT2/ZmMYBC1	Regulates anthocyanin accumulation.
ppa017136m	MYB	YL	PpTT2/ZmMYBC1	
ppa002884m	bHLH	HJ	PpbHLH3/AtTT8	Interacts with TT1, PAP1 and TTG1 on the regulation of flavonoid pathways.
ppa004070m	bHLH	HJ	PpPIF/AtPIF1	A key negative regulator of phytochrome-mediated seed germination, light-mediated suppression of hypocotyl elongation. Negatively regulates phyB mediated red light responses. Involved in shade avoidance response.
ppa017228m	bHLH	HJ/ YL	PpPIF/AtPIF4	
ppa010134m	bHLH	HJ/ YL	PpbHLH/AT2G40200	basic helix-loop-helix (bHLH) DNA-binding superfamily protein.
ppa006295m	bHLH	HJ/YL	PpbHLH130/AT1G51140	Involved in biological process of photoperiodism and flowering.
ppa011967m	bZIP	HJ	PpHYH/AtHYH	Involved in light signaling pathway.
ppa009024m	bZIP	HJ	ppa009024m/AtGBF4	Basic-leucine zipper (bZIP) transcription factor family protein.
ppa005585m	bZIP	YL	PpbZIP63/AtbZIP63	bZIP protein BZO2H3.
ppa009438m	NAC	HJ/YL	PpNAC2/AtNAC2	Transcript level increases in response to wounding and abscisic acid, attenuates ABA signaling and synthesis.
ppa009380m	NAC	HJ	PpNAC2/AtNAC2	
ppa026582m	NAC	HJ/YL	PpNAC68/AtNAC068	Interacts with VND7 and negatively regulates xylem vessel formation.
ppa007883m	NAC	HJ	PpNAC100/AtNAC100	NAC domain containing protein 100.
ppa025263m	NAC	YL	PpNAC42/AtNAC042	Involved in anthocyanin-containing compound biosynthetic process.


## Discussion

### Varied Sensitivity to UV Results in Differential Anthocyanin Accumulation

Anthocyanins are a group of natural pigments seen throughout the plant kingdom. However, the accumulation of anthocyanins varies greatly among plant species and varieties (Zoratti et al., 2014). Mutations in anthocyanin structural or regulatory genes are associated with the altered anthocyanin biosynthesis in some varieties. For example, a mutation in *SlDFR* is the cause for *anthocyanin without* phenotype of *aw* tomato (Goldsbrough et al., 1994); mutations of *VvMYBA1* results in loss of pigmentation in white grape cultivars (Kobayashi et al., 2004), and mutation of *MrMYB1* prevents anthocyanin accumulation in Chinese bayberry (Niu et al., 2010). In contrast, mutations in *MYB* promoter regions can result in increased gene expression and anthocyanin accumulation, such as *MdMYB10* in red-fleshed apple (Espley et al., 2009) and *Ruby* in blood oranges (Butelli et al., 2012).

Here we found that the peach cultivar ‘YL’ is naturally lightly colored. It is unlikely that a mutation in coding sequences of anthocyanin structural or regulatory genes causes this phenotype, since when the fruit are exposed to UVB anthocyanin accumulation is restored (**Figure 1**). Compared to ‘HJ,’ ‘YL’ is insensitive to UVA, as supported chemical and gene expression analysis (**Figures 1**, **4** and Supplementary Table S4), which may explain why ‘YL’ is lightly pigmented, since UVA accounts for over 95% of total UV energy in solar light.

The insensitivity of fruit to UVA is not commonly reported in fruits. However, in *Arabidopsis*, some mutants impaired in anthocyanin accumulation were reported to be associated with UVA/blue light insensitivity. A mutation in UVA/blue light receptor gene *AtCry1* or *AtHY5* can impair light-induced anthocyanin accumulation in germinating seedlings (Ahmad et al., 1995; Shin et al., 2013). The mechanisms involved for the UVA/blue light sensitivity might vary among plant species, and in peach, according to the gene expression data (**Figure 7**), the low expression levels of *CRYs* and *PHOT2* in ‘YL’ fruit appears to be one reason for insensitivity to UVA. The reasons for the low expression level of these genes might be related to a mutation in the gene promoter or alternatively in an upstream regulator gene, which needs further evaluation.

Sensitivity to UVB also varied between varieties. As observed in this study, ‘HJ’ is less sensitive to UVB than to UVA, and less sensitive to UVB than ‘YL’ in regard to anthocyanin biosynthesis (**Figures 1**, **4**). Varied sensitivity to UVB was also reported in another study by Scattino et al. (2014), who observed that UVB can increase the amount of anthocyanins accumulated in peel of ‘Suncrest’ and ‘Big Top’ peach while no anthocyanin accumulated in peel of ‘Babygold 7’ peach even following UVB exposure. At present, the mechanism for the different sensitivity of peach varieties to UVB was not revealed.

### Coordinated Expression of Genes at Multiple Levels was Responsible for UV Induced Anthocyanin Biosynthesis

For both cultivars, different expression levels of anthocyanin biosynthesis structural genes, especially late genes *DFR*, *ANS* and *UFGT*, resulted in differential anthocyanin accumulation (**Figure 4**). In addition, an anthocyanin transporter gene, *PpGST1* (ppa011307m), was found to be differentially expressed and co-ordinated up-regulated, coincident with anthocyanin accumulation (**Figure 4**). In *Arabidopsis*, the *GST* mutant *tt19* accumulates extremely low anthocyanin content and the importance of GSTs related to anthocyanin transport has also been reported in maize (Goodman et al., 2004), carnation (Larsen et al., 2003), petunia (Alfenito et al., 1998), and grape (Conn et al., 2008). The participation of *PpGST1* in anthocyanin accumulation under different UV-light needs to be further studied.

Plants use multiple photoreceptors, including PHYs, CRYs and PHOTs, and UVR8, to perceive specific wavelengths of light (red/far-red light, UVA/blue light, and UVB, respectively) (Rizzini et al., 2011; Casal, 2013; Li et al., 2013). Twelve light receptors, including one PHYA, three CRYs, two PHOTs, and six UVR8s were identified in peach (**Figure 7**). In our study, the transcription levels of red/far-red light photoreceptor (PHYA) decreased for both cultivars after UV-light treatment. The expression patterns of CRY2 and PHOT1 remarkably decreased and reached the lowest in ‘YL’ cultivar, which may be the reason why ‘YL’ was insensitive to UVA light. The six UVR8 genes transcriptionally differed and were not upregulated under UVB irradiation in both cultivars. In other species, light-induced gene expression changes can be observed within few hours (Cominelli et al., 2008; Li et al., 2010), so rapid changes in the expression levels of photoreceptors may have occurred before the first sampling point in peach.

HY5, a bZIP transcriptional regulator, is believed to be a central positive modulator for photomorphogenic responses downstream of photoreceptors (Lee et al., 2007). AtHY5 is found to promote anthocyanin accumulation by activating expression of anthocyanin pathway genes like *CHS* and *R2R3 MYBs* in response to light (Hardtke et al., 2000; Stracke et al., 2010). However, in this study, the abundance of *PpHY5* transcript was not directly correlated with the extent of anthocyanin accumulation in either cultivar. However, the expression values of *PpHYH*, a homologous gene of *HY5*, changed in parallel with the upregulation of the anthocyanin biosynthesis pathway structural genes. These results indicated that PpHYH, rather than PpHY5, might play a role in regulating anthocyanin biosynthesis in peach fruit.

Photomorphogenesis is suppressed by ubiquitin proteasome-mediated degradation. The ubiquitylation process consists of three sequential actions of an ubiquitin-activating enzyme (E1), an ubiquitin-conjugating enzyme (E2), followed by an ubiquitin-ligase enzyme (E3) (Wei et al., 1994). The RING-finger type ubiquitin E3 ligase COP1 is a central negative regulator of light signal transduction by altering its subcellular localization to interact with upstream light receptors and downstream target proteins, to control light-regulated plant development processes. In the dark, COP1 is localized in the nucleus, mediating photomorphogenesis by targeting TFs, such as MYB and HY5/HYH, for ubiquitination and degradation via the 26S proteasome pathway. Under light, expression of nuclear-localized transcription factor genes was promoted due to low abundance of COP1 in the nucleus (Lau and Deng, 2012). In apple skin, it has been revealed that anthocyanin levels are modulated by MdCOP1-mediated signaling, leading to activation and binding of MdHY5 to the promoter regions of *R2R3 MYBs* (Li et al., 2012). In peach the expression of *PpCOP1.1*, the most highly expressed member, was not differentially expressed in the UV-light treated fruit (**Figure 7** and Supplementary Table S5), while the transcriptional level of *PpCOP10* exhibited an opposite expression pattern with that of structural genes. COP10, an ubiquitin-conjugating enzyme (E2) variant (UEV), is also essential in photomorphogenesis (Sanch et al., 1998). AtCOP10 has been previously identified as a photomorphogenesis repressor for COP1-mediated degradation of transcription factor HY5 within the nucleus (Osterlund et al., 2000). Furthermore, COP10 can directly interact with DET1 and DDB1 to form COP10–DET1–DDB1 (CDD) complex, then along with COP1 and the COP9 signalosome (CSN) to promote the degradation of positive transcription factors of photomorphogenesis, such as HY5 and HYH, via the ubiquitin/26S proteasome system (Suzuki et al., 2002). In litchi, the expression level of *LcCOP10* gradually decreases after exposure to light (Zhang et al., 2016). In this study, PpCOP10 may be another component working with PpCOP1s in the ubiquitin-proteasome pathway to influence the coloration of peach peel.

Previous reports have demonstrated that TFs are critical for light-induced anthocyanin biosynthesis, such as MYBs in Chinese bayberry (Niu et al., 2010), apple (Li et al., 2012), litchi (Lai et al., 2014) and grape (Kobayashi et al., 2004). PpMYB10.1/10.2/10.3 and PpbHLH3, were reported to be regulators of anthocyanin biosynthesis in peach (Rahim et al., 2014), and were identified to correlate with UV-light induced anthocyanin accumulation in this study. In addition, another four candidate MYBs including ppa023812m and ppa017136m, whose *Zea mays* homolog (ZmMYBC1) is an activator of the anthocyanin pathway (Roth et al., 1991), were identified (**Table 1**). Three *PpbHLHs*, including two *PIFs* (ppa004070m, ppa017228m), were identified to be negatively correlated with anthocyanin biosynthetic genes. In addition to potential components of the MBW complex, NACs have been reported to be potentially related to the anthocyanin pathway. AtNAC078 regulates flavonoid biosynthesis under high light (Morishita et al., 2009). Here we observed five *NACs* with sensitivity to UVB light in both cultivars. BL and PpNAC1, identified to participate in the regulation of anthocyanin accumulation in peach flesh (Zhou et al., 2015), were not among the differentially expressed TF genes. Whether these candidate TFs are related to the regulation of anthocyanin metabolism, and the relationship between TFs and their target genes, needs to be further investigated.

**FIGURE 8:**
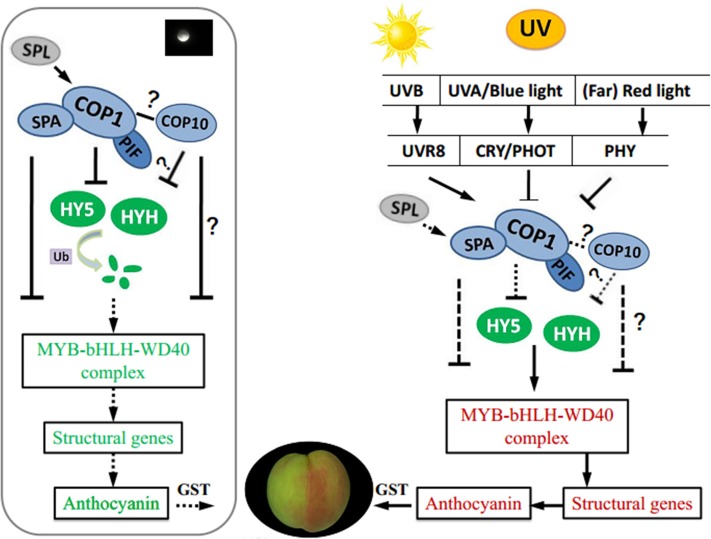
A proposed working hypothesis for UV-light-induced peach peel coloration. bHLH, basic helix–loop–helix; CRY, cryptochrome; COP1, constitutive photomorphogenic 1; COP10, constitutive photomorphogenic 10; GST, glutathione S-transferase; HY5, ELONGATED HYPOCOTYL 5; HYH, HY5 homolog; PHOT, phototropin; PHYA, phytochrome A; PIF, phytochrome-interacting basic helix-loop-helix transcription factors; SPA, suppressor of PHYA; SPL, SQUAMOSA promoter-binding protein-like; UVR8, UV RESISTANCE LOCUS 8; Ub, ubiquitin.

Based on transcriptome data, we analyzed identified candidate genes and TFs related to light-induced anthocyanin biosynthesis in UV-light treated peach. We proposed a working hypothesis of light-regulated anthocyanin biosynthesis in peach as a foundation for study in other plants (**Figure 8**). Coordinated expression of genes at multiple levels was responsible for UV induced anthocyanin biosynthesis. The negative regulators of photomorphogenesis, COP1 and COP10, together with PIF, SPA, SPL, can not only interact with upstream photoreceptors but also mediate the degradation of downstream light-response effectors, including HY5, HYH and members of the MBW complex, which regulate the transcription of anthocyanin biosynthesis structural genes. Further study is necessary to elucidate the mechanism of COP10 and HYH mediated effects on anthocyanin accumulation in peach.

## Author Contributions

YZ, BZ, and CX designed the research. YZ, WD, and KW performed the experiments; YZ, BZ, AA, KL-W, KC, and CX analyzed the data and wrote the manuscript. All of the authors read and approved the final manuscript.

## Conflict of Interest Statement

The authors declare that the research was conducted in the absence of any commercial or financial relationships that could be construed as a potential conflict of interest.

## Footnotes

1 http://www.rosaceae.org/peach/genome

2 http://www.genome.jp/kegg/

3 http://www.metaboanalyst.ca/MetaboAnalyst/faces/Secure/upload/StatUploadView.xhtml

4 https://www.ncbi.nlm.nih.gov/sra

5 http://www.genome.jp/kegg/
