# Supplementary material for: Differential Sensitivity of Fruit Pigmentation to Ultraviolet Light between Two Peach Cultivars
Source: Front Plant Sci. 2017 Sep 8;8:1552. doi: 10.3389/fpls.2017.01552 (PMC5596067; doi:10.3389/fpls.2017.01552)
Supplement: Supplementary file 1 [file Table_1.DOCX]

**Table S1 | Summary of the sequencing data and read mapping of ‘Hujingmilu’ (‘HJ’) and ‘Yulu’ (‘YL’) peach peel libraries.**

|  | Hujingmilu | | | Yulu | | |
| --- | --- | --- | --- | --- | --- | --- |
|  | **HJ_CK** | **HJ_UVA** | **HJ_UVB** | **YL_CK** | **YL_UVA** | **YL_UVB** |
| Number of raw reads | **14,939,389** | **13,476,997** | **14,447,921** | **48,798,599** | **43,163,902** | **45,538,605** |
| Number of clean reads | **14,925,811** | **13,457,867** | **14,437,226** | **47,688,624** | **42,069,214** | **44,479,700** |
| Clean bases (G) | **0.745** | **0.675** | **0.72** | **7.155** | **6.315** | **6.67** |
| GC content (%) | **45.77** | **46.23** | **46.485** | **45.905** | **45.925** | **46.15** |
| Q20 percentage (%) | **98.43** | **99.025** | **99.055** | **97.35** | **97.46** | **97.385** |
| Q30 percentage (%) | **96.82** | **98.095** | **98.16** | **93.03** | **93.285** | **93.115** |
| Total mapped (N,%) (percent of clean reads) | **14,234,894.5**  **(95.37%)** | **12,868,138**  **(95.625%)** | **13,893,941**  **(96.235%)** | **43,572,989**  **(91.38%)** | **37,548,144**  **(89.26%)** | **40,633,463**  **(91.33%)** |
| Multiple mapped (N,%)  (percent of clean reads) | **648,720.5**  **(4.345%)** | **534,977.5**  **(3.975%)** | **1,682,907.5**  **(11.655%)** | **699,796**  **(1.465%)** | **604,140.5**  **(1.435%)** | **1,230,077**  **(2.755%)** |
| Uniquely mapped (N,%)  (percent of clean reads) | **13,586,174**  **(91.025%)** | **12,333,160.5**  **(91.655%)** | **12,211,033.5**  **(84.58%)** | **42,873,193**  **(89.91%)** | **36,944,003.5**  **(87.825%)** | **39,403,386**  **(88.575%)** |
