# Supplementary material for: Differential Sensitivity of Fruit Pigmentation to Ultraviolet Light between Two Peach Cultivars
Source: Front Plant Sci. 2017 Sep 8;8:1552. doi: 10.3389/fpls.2017.01552 (PMC5596067; doi:10.3389/fpls.2017.01552)
Supplement: Supplementary file 2 [file Table_2.DOCX]

**Table S2 | Primers used for quantitative real-time PCR analysis of anthocyanin biosynthetic and regulatory genes in peach.**

| **Gene** | **Forward primer (5′ to 3′ )** | **Reverse primer (5′ to 3′ )** | **Phytozome/GenBank no.** |
| --- | --- | --- | --- |
| *PpPAL* | TTGCCATGGATAACACCAG | GATTTGAAGGCAACCCATTG | ppa002328m |
| *PpCHS* | CAGAGATACCCAAAGGTTGGAAGGC | AACCATCCTTCCCGACAGCGAT | ppa006888m |
| *PpCHI* | TGAAGACCTCAAGGAACTTCTCAATGG | ACACAGGTGACAACGATACTGCCACT | ppa011276m |
| *PpF3H* | TCCGAGGGCAGAGCGAAGAAC | TTGTGGAGGCTTGTGAGGATTGG | ppa007636m |
| *PpF3’H* | CCCAACTTGACCTACCTCCA | CTTTGGGATGTGGAAGCTGT | ppa004433m |
| *PpDFR* | GGTCGTCCAGGTGAACATACTGCC | ATTTCTCATGCCATCCATGCCAC | ppa008069m |
| *PpANS* | AAGTGGGTCACTGCCAAGTGTGTTC | GTGGCTCACAGAAAACTGCCCAT | ppa007738m |
| *PpUFGT* | CCGCTGCCTCTCCCAACACTC | CCATCAGCCACATCAAACACCTTTAT | ppa005162m |
| *PpMYB10.1* | CAGGAAGGACAGCGAATGATG | TCGGGGTTGAGGTCTTATTACG | ppa026640m |
| *PpMYB10.2* | TGATTCCAAGGGTCCACGCTAAAA | CTGGTCTTGGGTTAGATGAAGAACTGC | ppa016711m |
| *PpMYB10.3* | GTACGCCATCACAAACATCACC | CATCATCAACCCAAAAACTCGT | ppa020385m |
| *PpbHLH3* | TTCCTCTACTAGACGGCGTCGTCG | GGAGGAGGATGGTGGTTGTGGTC | ppa002884m |
| *PpWD40-1* | CCCAGCCTGATACCCCTTTGCT | GTCGGCGAACGGATATCCAAAAT | ppa008187m |
| *PpTEF2 (Actin)* | GGTGTGACGATGAAGAGTGATG | TGAAGGAGAGGGAAGGTGAAAG | JQ732180 |
