# Supplementary material for: Differential Sensitivity of Fruit Pigmentation to Ultraviolet Light between Two Peach Cultivars
Source: Front Plant Sci. 2017 Sep 8;8:1552. doi: 10.3389/fpls.2017.01552 (PMC5596067; doi:10.3389/fpls.2017.01552)
Supplement: Supplementary file 3 [file Table_3.DOCX]

**Table S3 | The real-time PCR value and FPKM value for the selected genes.**

| **Gene** | **X=log2（FPKM)** | **Y= QPCR** |
| --- | --- | --- |
| HJ_UVA CHS | 8.160 | 1.064 |
| HJ_UVB CHS | 9.381 | 2.379 |
| HJ_CK CHI | 5.540 | 0.132 |
| HJ_UVA CHI | 6.372 | 0.281 |
| HJ_CK F3H | 5.179 | 0.130 |
| HJ_CK ANS | 9.422 | 1.156 |
| HJ_UVA ANS | 11.069 | 3.989 |
| HJ_UVB ANS | 9.484 | 2.783 |
| HJ_UVB UFGT | 6.105 | 0.110 |
| HJ_CK WD40-1 | 5.741 | 0.125 |
| HJ_UVA WD40-1 | 6.457 | 0.148 |
| YL-UVB CHS | 5.600 | 0.126 |
| YL-CK CHI | 6.200 | 0.198 |
| YL-UVA CHI | 6.273 | 0.237 |
| YL-UVB CHI | 7.521 | 0.463 |
| YL-CK F3H | 4.973 | 0.058 |
| YL-UVB F3H | 6.385 | 0.141 |
| YL-CK DFR | 4.990 | 0.004 |
| YL-UVA DFR | 4.504 | 0.004 |
| YL-UVA ANS | 9.556 | 1.454 |
| YL-UVB ANS | 10.093 | 2.332 |
| YL-CK BHLH3 | 5.431 | 0.040 |
| YL-UVA BHLH3 | 5.394 | 0.041 |
| YL-UVB BHLH3 | 4.987 | 0.035 |
| YL-CK WD40-1 | 5.264 | 0.038 |
| YL-UVA WD40-1 | 5.331 | 0.042 |
| YL-UVB WD40-1 | 6.127 | 0.052 |
