# Supplementary material for: Differential Sensitivity of Fruit Pigmentation to Ultraviolet Light between Two Peach Cultivars
Source: Front Plant Sci. 2017 Sep 8;8:1552. doi: 10.3389/fpls.2017.01552 (PMC5596067; doi:10.3389/fpls.2017.01552)
Supplement: Supplementary file 4 [file Table_4.DOCX]

**Table S4 |** **Expression profiles (FPKM) of anthocyanin biosynthesis structural and regulatory genes.**

|  | **geneID** | **HJ_CK** | **HJ_UVA** | **HJ_UVB** | **YL_CK** | **YL_UVA** | **YL_UVB** |
| --- | --- | --- | --- | --- | --- | --- | --- |
| PAL1 | ppa002328m | 0.15 | 0.24 | 1.94 | 1.73 | 0.87 | 4.35 |
| PAL2 | ppa002099m | 1.91 | 27.40 | 91.51 | 2.41 | 0.56 | 14.53 |
| C4H | ppa004544m | 34.26 | 90.35 | 38.57 | 14.76 | 12.96 | 18.11 |
| 4CL1 | ppa003893m | 42.64 | 60.88 | 68.19 | 14.13 | 12.74 | 20.81 |
| 4CL2 | ppa019293m | 4.84 | 6.65 | 13.10 | 0.00 | 0.00 | 0.00 |
| 4CL3 | ppa003871m | 33.40 | 31.83 | 17.41 | 42.12 | 37.15 | 40.27 |
| 4CL4 | ppa003506m | 195.65 | 234.90 | 548.43 | 117.54 | 100.59 | 226.30 |
| 4CL5 | ppa003742m | 23.98 | 26.21 | 11.26 | 30.82 | 30.59 | 21.81 |
| 4CL6 | ppa022401m | 15.99 | 53.53 | 76.14 | 8.86 | 7.73 | 25.07 |
| 4CL7 | ppa003747m | 1.44 | 1.33 | 2.44 | 4.83 | 1.81 | 2.51 |
| 4CL8 | ppa003658m | 42.93 | 50.69 | 111.83 | 36.70 | 28.50 | 39.79 |
| CHS1 | ppa006888m | 14.94 | 285.98 | 666.96 | 7.33 | 1.89 | 48.51 |
| CHS2 | ppa006899m | 4.94 | 83.08 | 67.60 | 9.93 | 1.79 | 21.26 |
| CHS3 | ppa008402m | 12.60 | 187.03 | 45.53 | 10.80 | 2.89 | 25.47 |
| CHS4 | ppa023080m | 1.12 | 22.58 | 101.66 | 2.58 | 0.41 | 8.55 |
| CHI1 | ppa011276m | 46.52 | 82.83 | 236.95 | 73.49 | 77.31 | 183.70 |
| CHI2 | ppa011476m | 8.59 | 32.52 | 28.02 | 14.26 | 6.79 | 33.93 |
| F3H | ppa007636m | 36.23 | 225.09 | 186.28 | 31.42 | 9.69 | 83.59 |
| F3’H | ppa004433m | 304.49 | 847.16 | 381.45 | 242.48 | 119.49 | 201.39 |
| DFR1 | ppa008069m | 23.88 | 190.11 | 97.15 | 31.79 | 22.69 | 86.59 |
| DFR2 | ppa008011m | 32.58 | 46.28 | 13.52 | 52.97 | 62.82 | 31.30 |
| ANS | ppa007738m | 685.85 | 2148.04 | 716.23 | 378.99 | 752.85 | 1092.51 |
| UFGT | ppa005162m | 20.77 | 321.37 | 68.82 | 6.31 | 6.94 | 18.69 |
| MYB10.1 | ppa026640m | 2.59 | 14.46 | 24.96 | 1.86 | 0.61 | 1.82 |
| MYB10.2 | ppa016711m | 0.00 | 0.00 | 7.97 | 0.00 | 0.00 | 0.58 |
| MYB10.3 | ppa020385m | 0.00 | 0.00 | 6.83 | 0.00 | 0.00 | 0.28 |
| BHLH3 | ppa002884m | 43.85 | 50.58 | 12.85 | 43.15 | 42.04 | 31.71 |
| WD40-1 | ppa008187m | 53.47 | 87.88 | 144.28 | 38.44 | 40.26 | 69.91 |
| GST1 | ppa011307m | 11.22 | 144.63 | 251.57 | 0.76 | 0.76 | 38.57 |
