# Supplementary material for: Differential Sensitivity of Fruit Pigmentation to Ultraviolet Light between Two Peach Cultivars
Source: Front Plant Sci. 2017 Sep 8;8:1552. doi: 10.3389/fpls.2017.01552 (PMC5596067; doi:10.3389/fpls.2017.01552)
Supplement: Supplementary file 5 [file Table_5.DOCX]

**Table S5 | Expression profiles (FPKM) of photoreceptors and light signal transduction elements.**

|  | geneID | HJ_CK | HJ_UVA | HJ_UVB | YL_CK | YL_UVA | YL_UVB |
| --- | --- | --- | --- | --- | --- | --- | --- |
| PHYA | ppa000643m | 50.579 | 44.924 | 9.175 | 40.615 | 41.447 | 31.546 |
| CRY1 | ppa002375m | 13.811 | 19.124 | 43.865 | 17.675 | 17.480 | 22.647 |
| CRY2 | ppa002669m | 64.214 | 51.342 | 34.275 | 52.323 | 38.162 | 41.606 |
| CRY3 | ppa003875m | 1.785 | 19.882 | 12.756 | 4.273 | 3.466 | 3.419 |
| PHOT1 | ppa000777m | 40.851 | 46.276 | 16.710 | 41.853 | 27.605 | 26.794 |
| PHOT2 | ppa000797m | 0.617 | 2.383 | 3.544 | 1.512 | 1.502 | 1.507 |
| UVR8.1 | ppa005822m | 76.883 | 95.267 | 17.854 | 68.907 | 45.602 | 30.891 |
| UVR8.2 | ppa005731m | 11.875 | 4.793 | 4.134 | 4.084 | 4.742 | 6.958 |
| UVR8.3 | ppa004014m | 23.833 | 16.419 | 8.531 | 96.069 | 82.379 | 73.315 |
| UVR8.4 | ppa020628m | 3.160 | 2.756 | 0.728 | 2.044 | 2.657 | 1.478 |
| UVR8.5 | ppa016519m | 2.350 | 2.604 | 0.705 | 4.418 | 3.707 | 2.780 |
| UVR8.6 | ppa004744m | 43.808 | 44.676 | 21.390 | 29.842 | 36.001 | 30.144 |
| HY5 | ppa012064m | 27.330 | 12.873 | 34.836 | 53.438 | 25.101 | 40.961 |
| HYH | ppa011967m | 0.466 | 16.330 | 18.283 | 0.690 | 0.802 | 3.156 |
| COP1.1 | ppa002554m | 21.887 | 24.318 | 29.735 | 29.092 | 28.214 | 30.569 |
| COP1.2 | ppa002635m | 0.133 | 1.780 | 0.331 | 0.504 | 0.377 | 0.645 |
| COP10 | ppa012102m | 94.391 | 60.665 | 44.433 | 76.405 | 93.149 | 65.370 |
| SPA1 | ppa014569m | 3.846 | 8.424 | 5.800 | 12.337 | 8.353 | 8.486 |
| SPA2 | ppa000607m | 3.832 | 7.852 | 6.363 | 8.359 | 5.559 | 6.505 |
| SPA3 | ppa001120m | 3.268 | 7.726 | 14.802 | 6.395 | 4.905 | 6.358 |
| PIF1 | ppa004070m | 1.859 | 1.418 | 0.442 | 1.386 | 1.282 | 0.698 |
| PIF2 | ppa017228m | 1.792 | 0.634 | 0.456 | 1.873 | 1.515 | 0.244 |
| PIF3 | ppa001899m | 9.655 | 10.692 | 27.831 | 11.862 | 7.880 | 13.261 |
| SPL1 | ppa000690m | 76.992 | 101.064 | 93.795 | 102.135 | 85.463 | 88.003 |
| SPL2 | ppa005013m | 4.608 | 2.671 | 9.309 | 5.042 | 4.752 | 7.950 |
| SPL3 | ppa017695m | 13.580 | 9.349 | 0.357 | 4.858 | 4.733 | 2.313 |
| SPL4 | ppa023657m | 9.463 | 10.579 | 13.063 | 18.792 | 14.433 | 17.406 |
| SPL5 | ppa007202m | 67.863 | 38.687 | 31.766 | 74.629 | 91.749 | 63.602 |
| SPL6 | ppa000682m | 33.145 | 59.095 | 42.285 | 40.183 | 39.414 | 42.276 |
| SPL7 | ppa003644m | 6.806 | 5.559 | 2.955 | 2.712 | 2.293 | 2.287 |
