# Supplementary figures and images for: Differential Sensitivity of Fruit Pigmentation to Ultraviolet Light between Two Peach Cultivars
Source: Front Plant Sci. 2017 Sep 8;8:1552. doi: 10.3389/fpls.2017.01552 (PMC5596067; doi:10.3389/fpls.2017.01552)

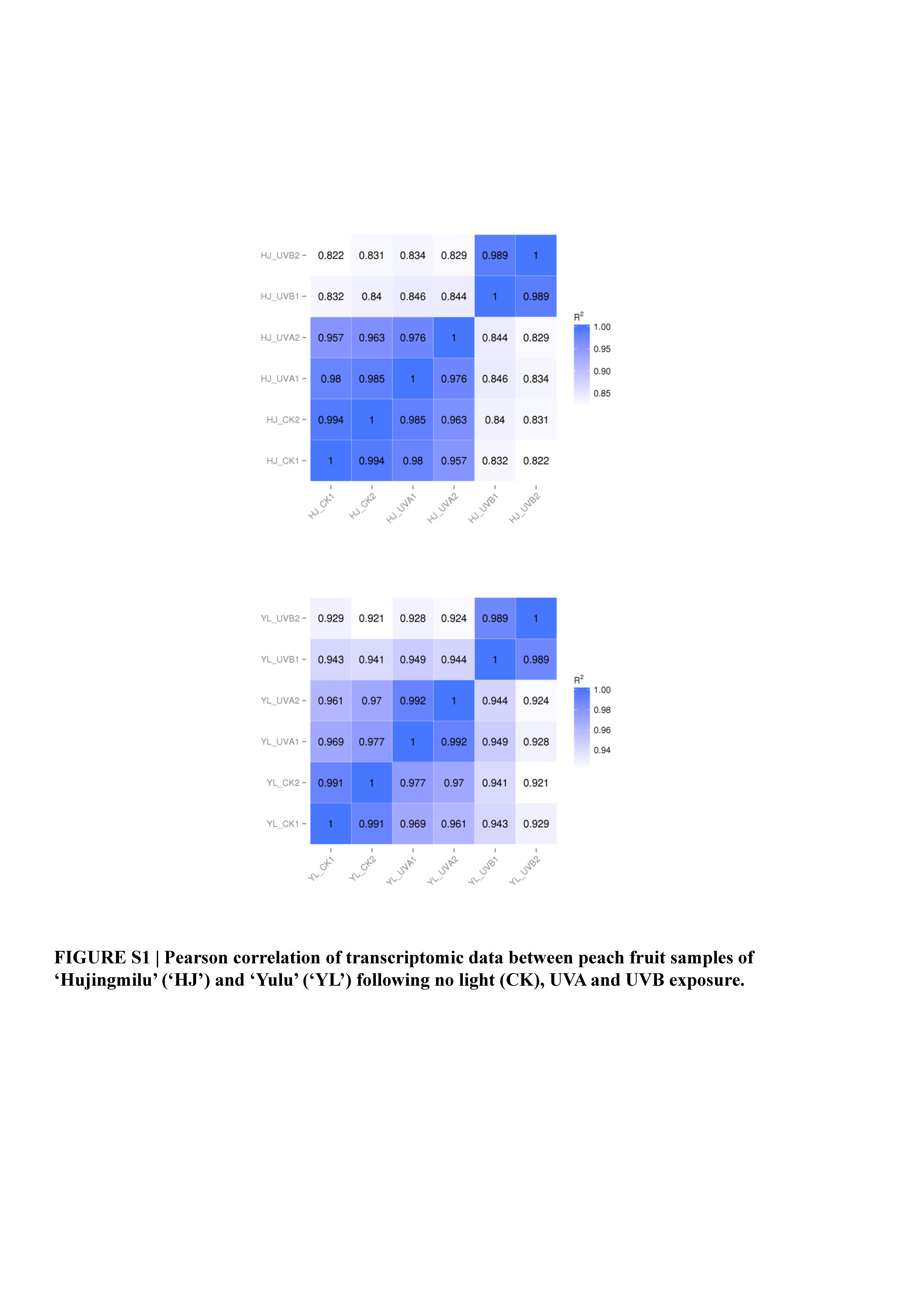

Supplement: Supplementary file 6 [file Image_1.JPEG]

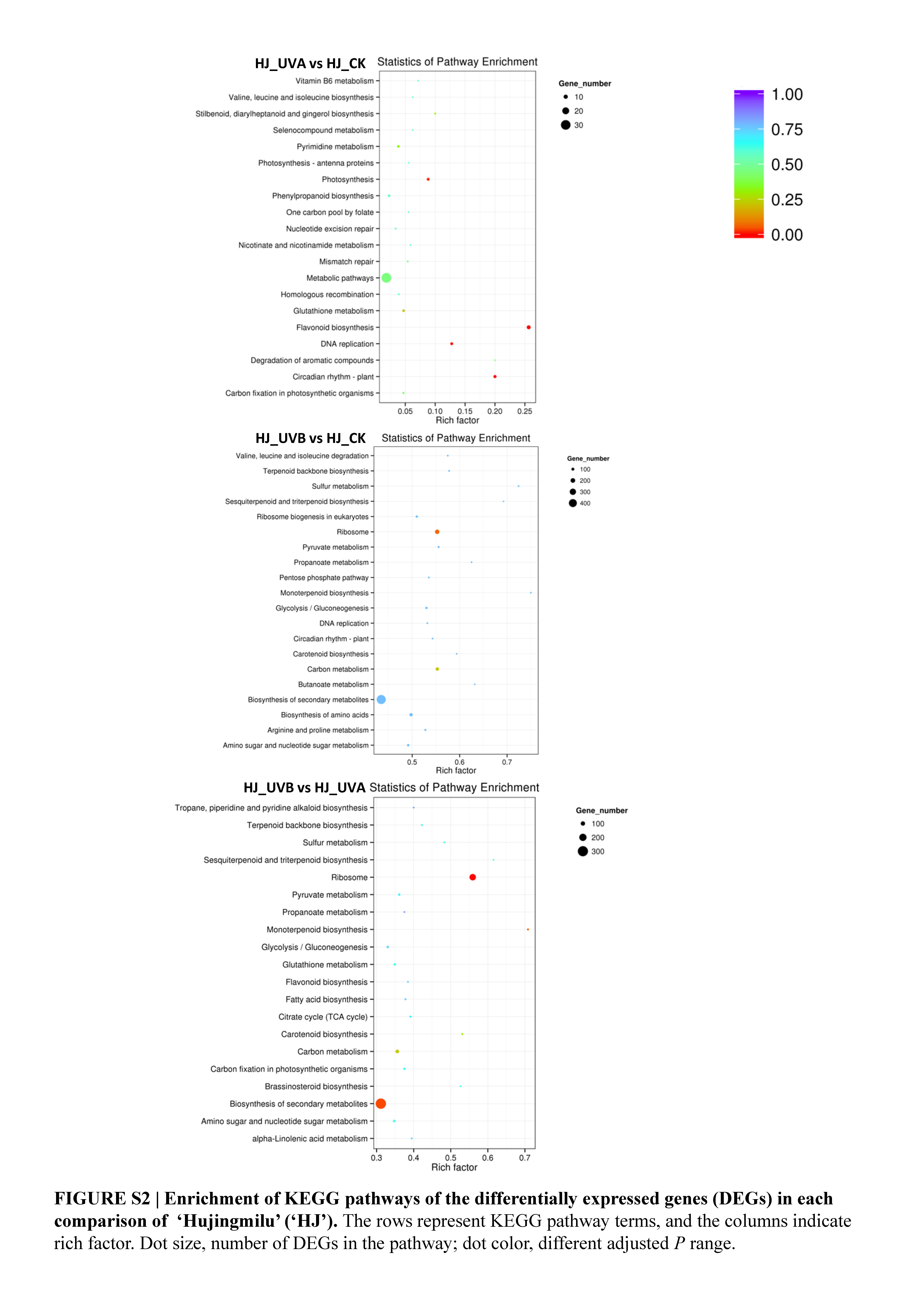

Supplement: Supplementary file 7 [file Image_2.JPEG]

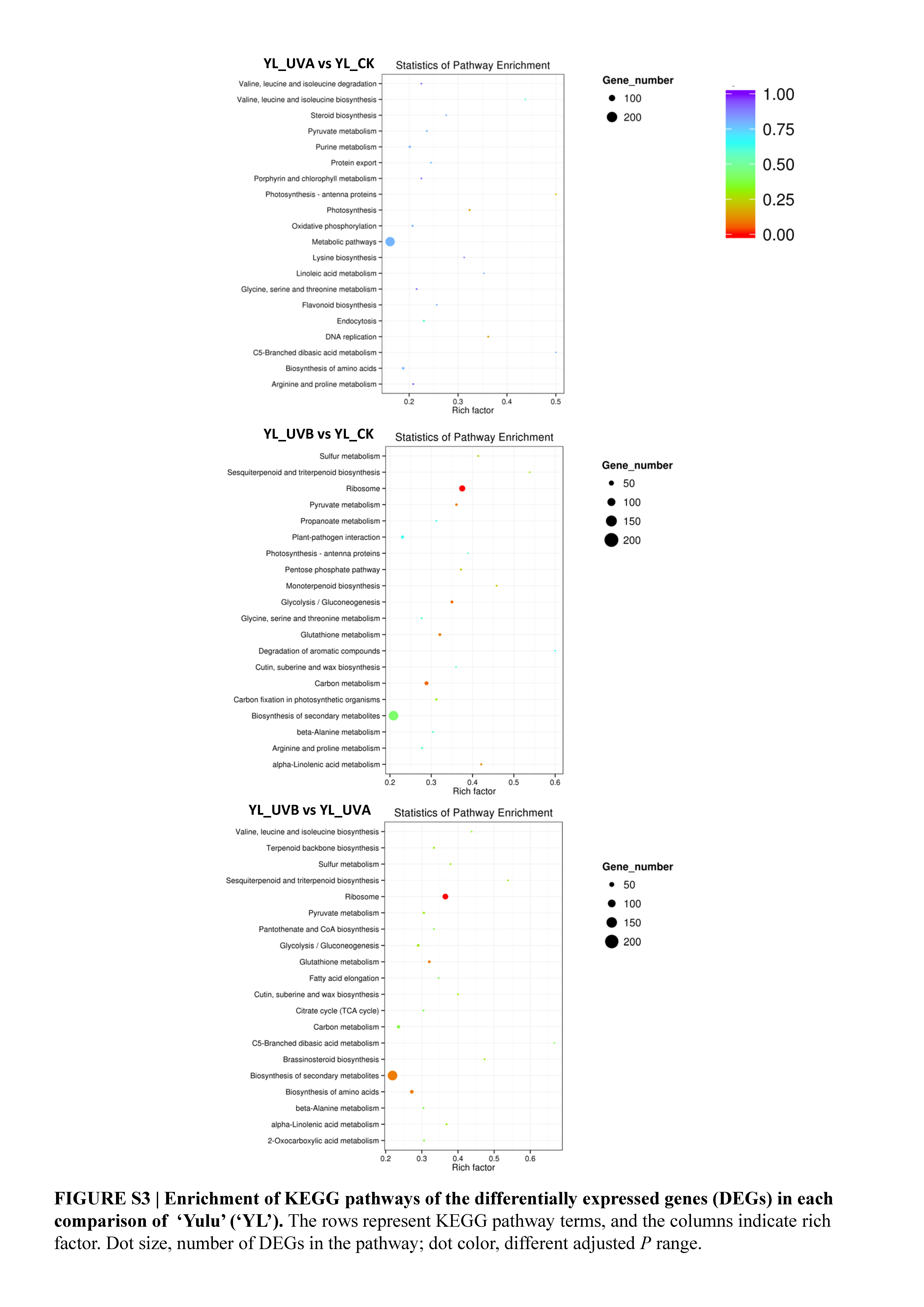

Supplement: Supplementary file 8 [file Image_3.JPEG]

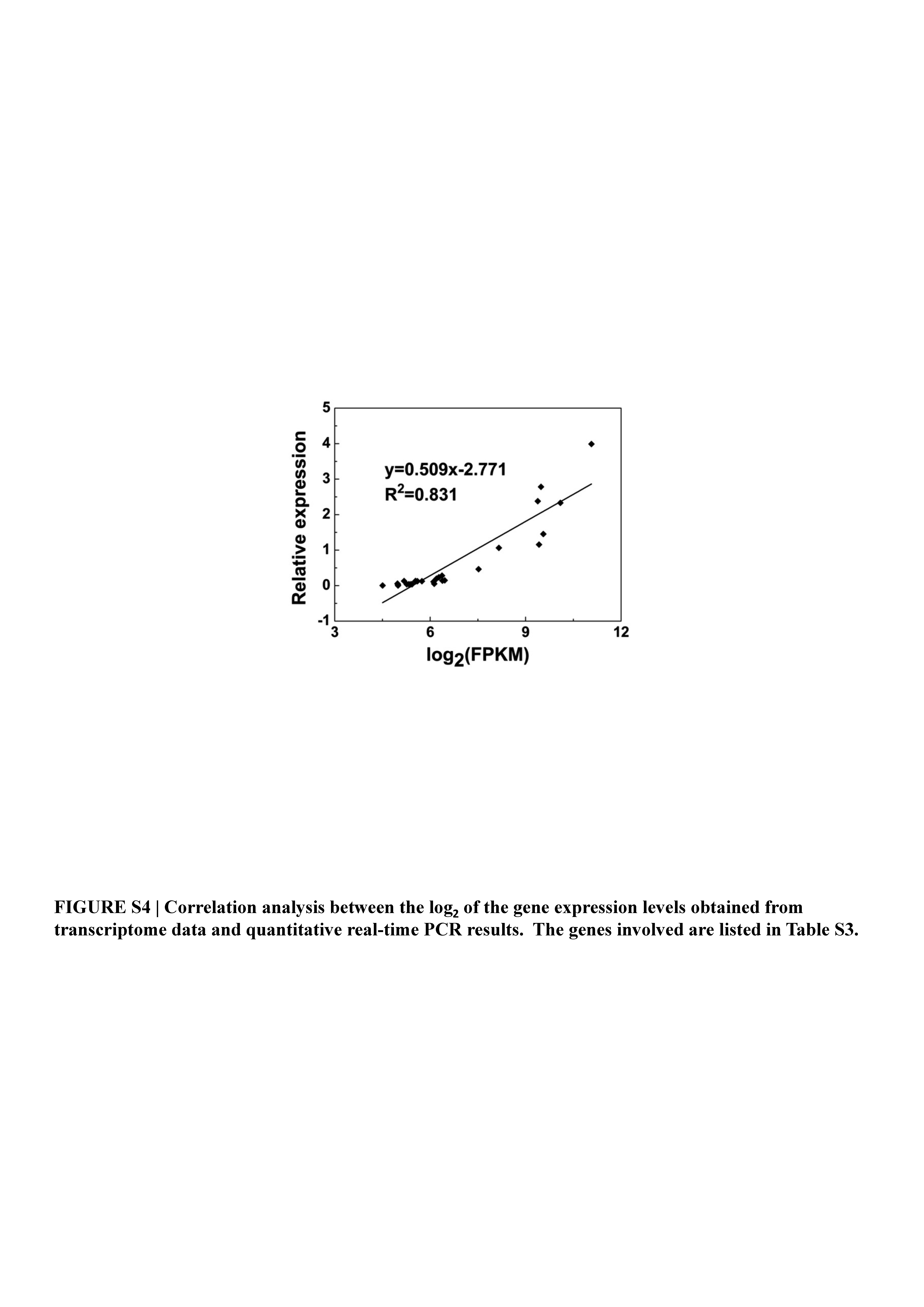

Supplement: Supplementary file 9 [file Image_4.JPEG]

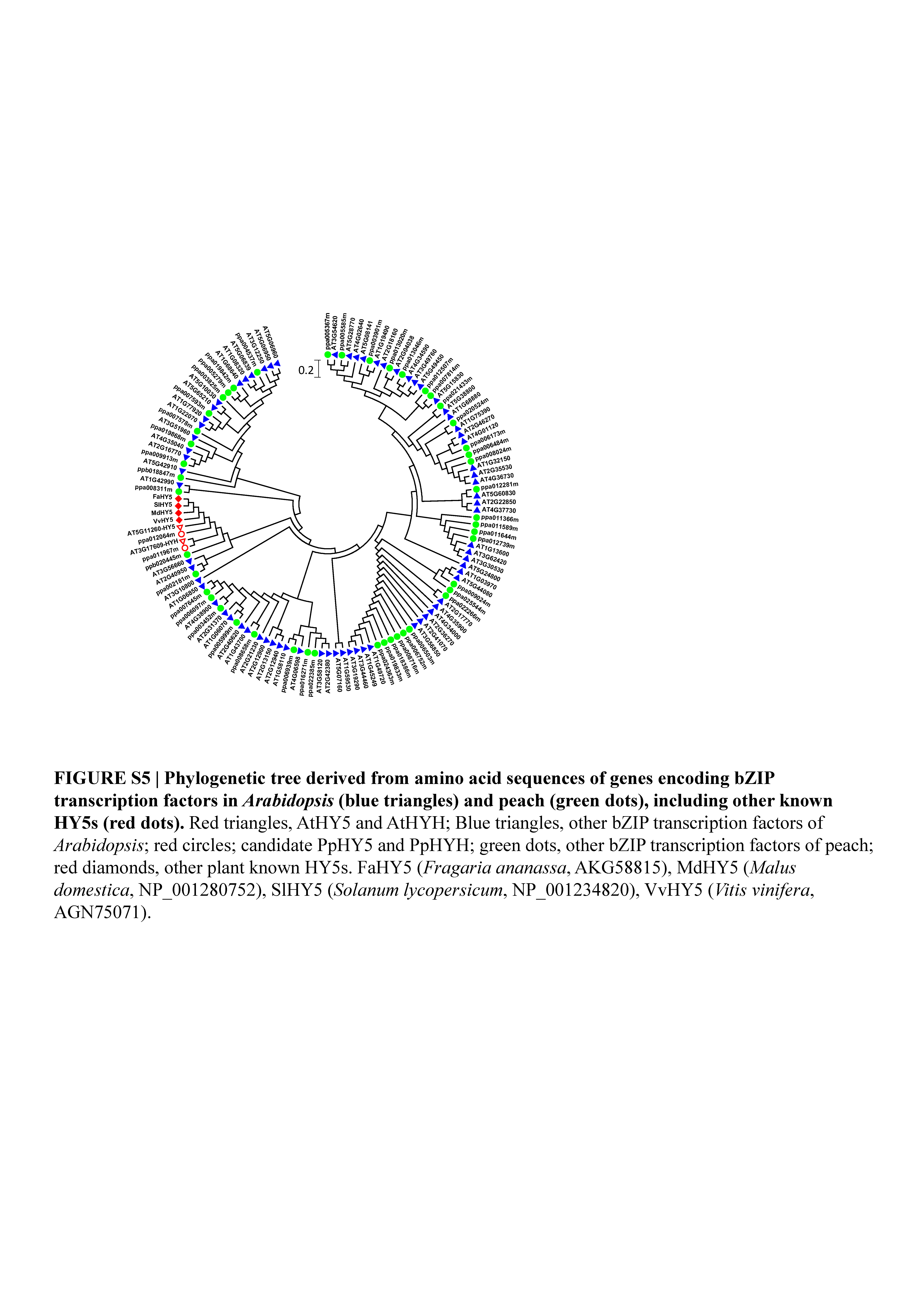

Supplement: Supplementary file 10 [file Image_5.jpg]

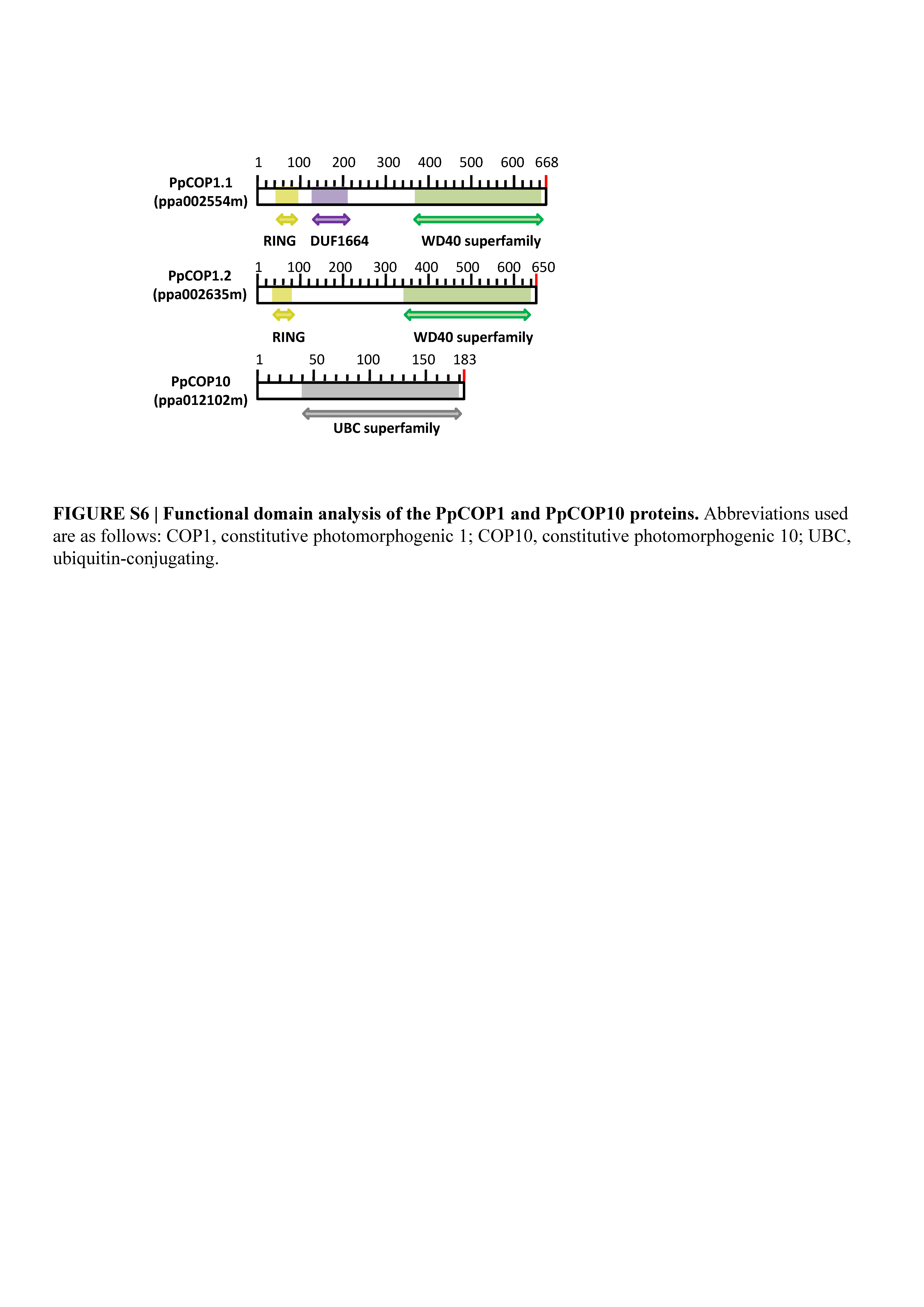

Supplement: Supplementary file 11 [file Image_6.jpg]

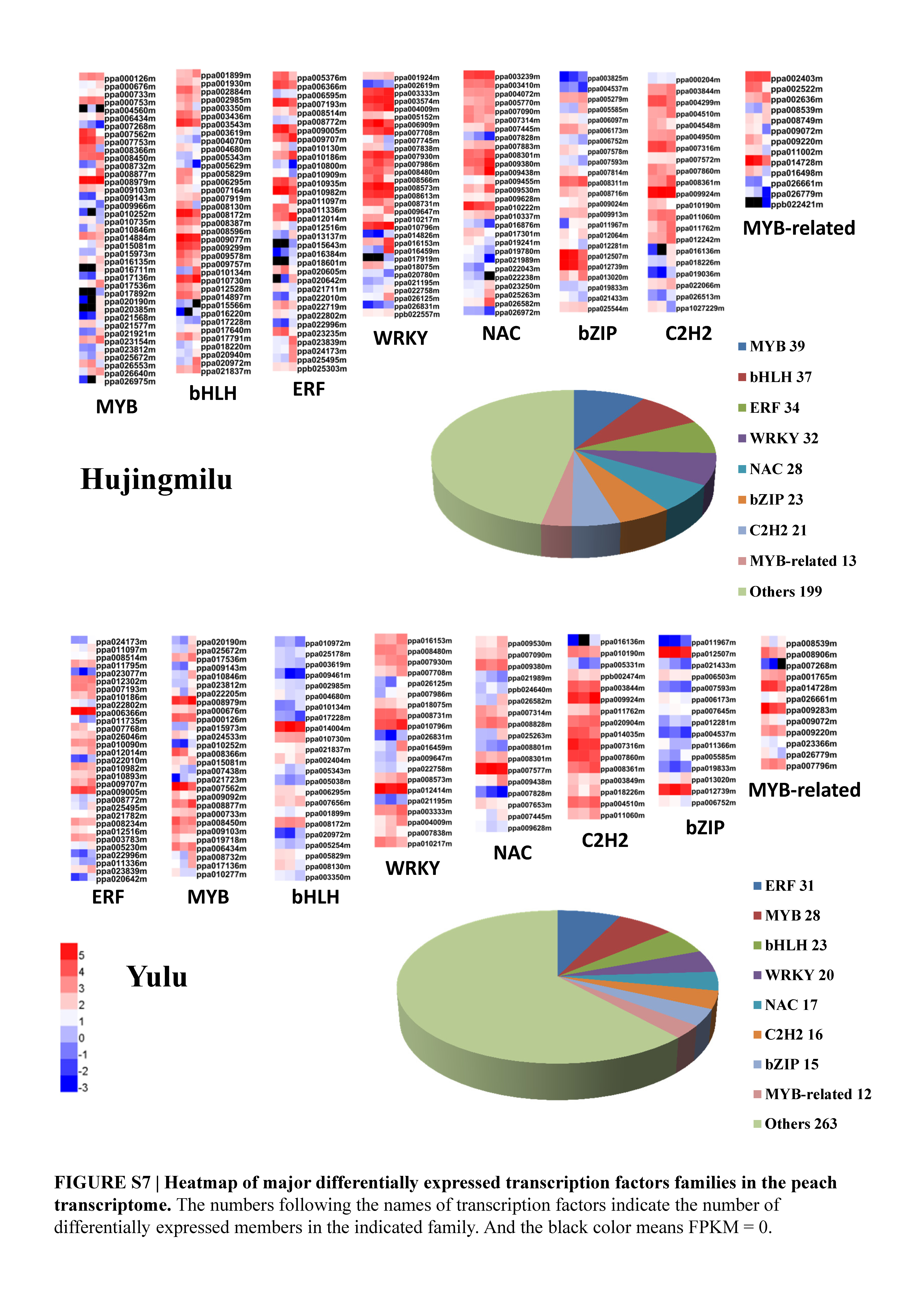

Supplement: Supplementary file 12 [file Image_7.jpg]
